# Monitoring to detect changes in water quality to meet policy objectives

**DOI:** 10.1038/s41598-024-52512-7

**Published:** 2024-01-22

**Authors:** R. W. McDowell, A. Noble, M. Kittridge, O. Ausseil, C. Doscher, D. P. Hamilton

**Affiliations:** 1 AgResearch, Lincoln Science Centre, Lincoln, New Zealand; 2 Faculty of Agriculture and Life Sciences, Lincoln University, Lincoln, New Zealand; 3 Headwaters Hydrology, Christchurch, New Zealand; 4 Traverse Environmental, Wellington, New Zealand; 5 Australian Rivers Institute, Griffith University, Queensland, Australia

**Keywords:** Environmental sciences, Hydrology, Limnology

## Abstract

Detecting change in water quality is key to providing evidence of progress towards meeting water quality objectives. A key measure for detecting change is statistical power. Here we calculate statistical power for all regularly (monthly) monitored streams in New Zealand to test the effectiveness of monitoring for policy that aims to decrease contaminant (phosphorus and nitrogen species, *E. coli* and visual clarity) concentrations to threshold levels in 5 or 20 years. While > 95% of all monitored sites had sufficient power and samples to detect change in nutrients and clarity over 20 years, on average, sampling frequency would have to double to detect changes in *E. coli.* Furthermore, to detect changes in 5 years, sampling for clarity, dissolved reactive phosphorus and *E. coli* would have to increase up to fivefold. The cost of sampling was predicted to increase 5.3 and 4.1 times for 5 and 20 years, respectively. A national model of statistical power was used to demonstrate that a similar number of samples (and cost) would be required for any new monitoring sites. Our work suggests that demonstrating the outcomes of implementing policy for water quality improvement may not occur without a step change in investment into monitoring systems. Emerging sampling technologies have potential to reduce the cost, but existing monitoring networks may also have to be rationalised to provide evidence that water quality is meeting objectives. Our study has important implications for investment decisions involving balancing the need for intensively sampled sites where changes in water quality occur rapidly versus other sites which provide long-term time series.

## Introduction

Water quality monitoring regimes are designed to indicate the state and trend of contaminants in streams and rivers, to align with catchment objectives and water quality policy. If either state or trend is considered undesirable (i.e., exceeding water quality thresholds) where agricultural land use contributes to contaminant inputs, farm management actions are suggested (or mandated) to reduce the loss of sediment and faecal bacteria (o their indicators) and nutrients to rivers^1^. Depending on the magnitude and speed of change required, the stock of contaminants in the river and the sampling regime, it can take many years to prove that an objective has been achieved^2,3^.

Robust monitoring regimes rely on capturing enough samples, over a long-enough period, to increase the likelihood (power) of detecting a significant (*P* < 0.05) magnitude of desired change^4^. However, while factors like ease of access, spatial representativeness, and the sensitivity of the river to contamination are commonly considered, statistical power is not. Statistical power is the probability that the expected result (e.g., a change in contaminant concentration) is real. Although more recent work assess the likelihood of an effect by credibility intervals^5^, it is still commonly, to use a binary threshold for statistical power of ≥ 0.80 as the level at which the likelihood of an effect (one-sided test) is real^6^, but this is influenced by the critical level (ɑ), sample size, the desired level of effect (e.g., percentage reduction in the median of a water quality measure) and variability in the data. If the critical level is set at *P* < 0.05, we can use the variability (standard deviation of the contaminant concentrations distributed around a point in time, hereafter termed—standard deviation) in the data to calculate either the level of reduction detectable with a specified number of samples or the number of samples required to meet a specified level of reduction. This helps those involved in implementing water quality policy avoid missing the opportunity to detect an effect and coming to the wrong conclusion, but it also helps forecast the time it may take to detect the change and the cost of detection since our main method of increasing power is to increase the number of samples taken^7^.

Collecting and analysing water samples is costly^8^, meaning that most sampling regimes will take samples at frequencies ranging from fortnightly to quarterly, with monthly being most common^9^. Regulators try to remove bias in these regimes associated with diurnal cycles and the availability of staff by scheduling sampling to occur at the same time of day and during the work week. However, at that frequency it can take many years to detect progress towards or achievement of, policy targets, such as a desired percentage reduction in contaminant concentrations. For instance, it was estimated that at the current sampling rate across 13 Canadian catchments, detecting a policy target of a 40% reduction in total phosphorus^10^ would take 8–50 years of data^4^. In another example, the New Zealand Government has set a desire in policy to see improvements in water quality within five years^11^, but a preliminary analysis of 77 catchments of the National River Water Quality Network (with 30 years of monthly data) suggested that changes in nitrate–N (NO_3_-N) would be difficult to detect within 10 years^12^. However, it is important to realise that the catchments within this network are large (mean size 2640 km^2^) and that changes would probably be detected faster in smaller catchments where monitoring sites were closer to where land management actions occurred^13,14^.

Information is clearly required on the likely performance of existing monitoring programmes (being a network of sites monitored at a given frequency) to detect change but also on the likelihood for new or modified monitoring programmes. However, data to derive medians and standard deviations for contaminant concentrations that are representative of the entire stream network are seldom available. Large networks with sites that exhibit strong changes in concentrations can overcome some of the deficiencies associated with poor spatial coverage and through modelling can provide statistical approximations of stream concentrations^15^. For instance, previous work has used national or international classifications to group catchment characteristics in models among sites and use these classes to extend predictions to areas where no data are available. Dodds and Oakes^16^ classified reference concentrations for nutrients across the continental US by the catchment characteristics encapsulated by the Ecoregion approach^17^. In New Zealand, the River Environment Classification classifies rivers and catchments according to factors like climate, topography and geology and has been successfully used to predict nutrient concentrations^18^, hydrological flows^19^, reference conditions^20^, and algal growth^21^. These approaches have focused on concentrations and flux estimates, but no work has focused on estimating the standard deviations necessary for power calculations.

Our aim was to derive estimates of the likelihood of detecting change at sites in a large national dataset and use these data to test two scenarios. The first scenario output the mean number of samples and costs required to detect an improvement in river water quality (reduce contaminant concentrations and turbidity, increase visual clarity) to national bottom lines (i.e. minimum acceptable state) in the New Zealand Government’s National Policy Statement for Freshwater Management (NPS-FM)^11^ and maintain current sampling regimes (where not exceeding bottom lines) in monitored sites within a five- and 20-year period. The timing is commensurate with New Zealand government policy to start making improvements within five year and bring waterways to a healthy state within a generation^22^. The second scenario output the mean number of samples and cost needed to detect a change of 30% improvement in current river water quality within a five- and 20-year period at all monitoring sites. This reduction equates to the mean reductions possible with the implementation of strategies to mitigate nitrogen and phosphorus losses from land to water by 2035^23^. This policy is intended to protect ecosystem health in rivers and in lakes but is set via concentrations. Because few of the monitored sites flow into lakes, we only used national bottom lines pertaining to rivers.

We also combined sampling data with predictor variables from existing classifications in the first national model to predict the likelihood of detecting change in riverine contaminant concentrations nationally. This model can be used to guide decisions about further investigations to determine where and at what cost new sites can detect changes in the concentration of a range of water quality contaminants.

## Results

**Figure 1:**
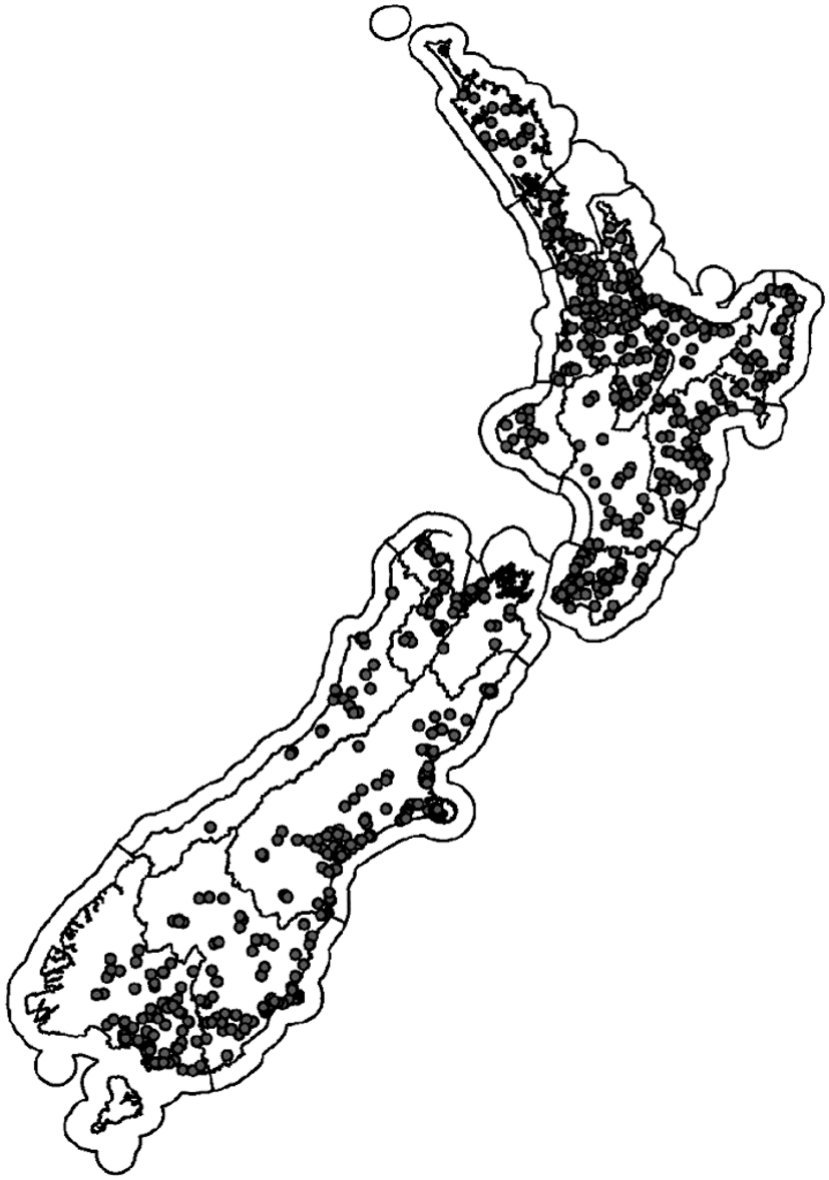
Location of sampling sites (after data were filtered) within New Zealand by region. Being a mountainous, but narrow country, relatively few higher stream orders drain into inland lakes than many other jurisdictions.

**Figure 2:**
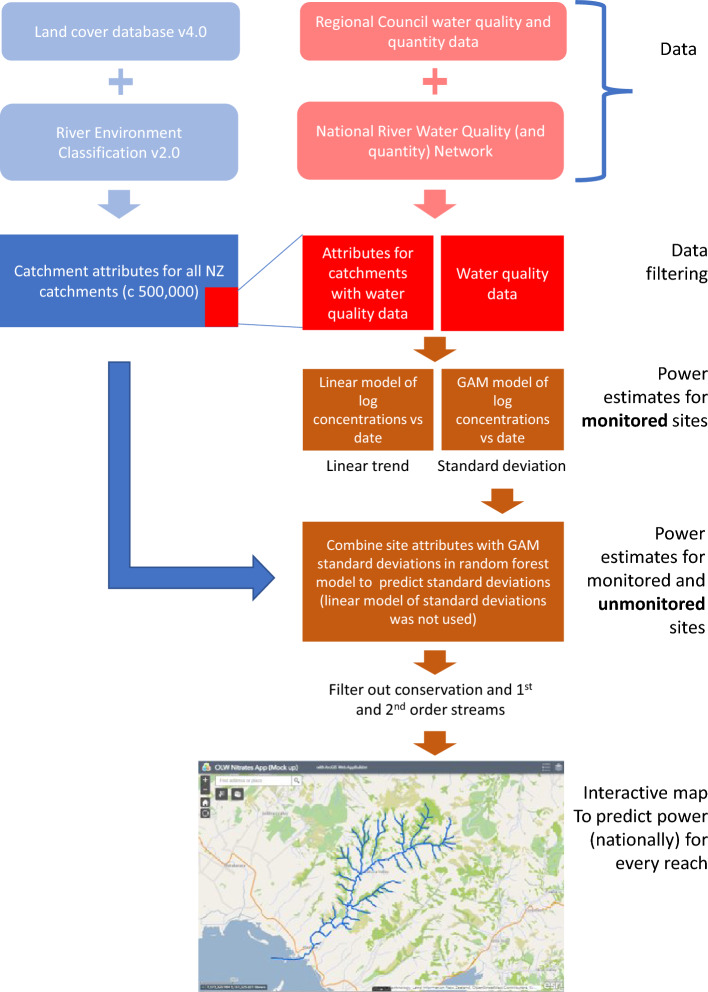
Flow diagram showing the filtering and modelling of data into the interactive map.

The following sections outline our efforts to filter the data so that sites (Fig. 1) with observations are more representative of the national river network. This process (Fig. 2) produced data filtered to estimate the likelihood of detecting a change in concentration and monitoring costs associated with the two scenarios described above. Finally, we describe the performance of the national model that can be used to predict the likelihood of detecting change in unmonitored sites.

### Existing monitoring data representativeness

Median values for 17 continuous largely local or catchment scale climatic and land use variables (Table S1) were different between the ~ 550,000 reaches in the stream network compared to those reaches with monitoring sites on them. However, once first and second order streams (n ~ 400,000) were filtered out, only nine variables had different median values (Table S2). A similar pattern occurred with categorical REC classes with eight out of 31 classes having different proportions between the whole network and monitored sites, reducing to six classes once filtered (Table S3).

We can conclude that filtering the data made the monitored sites more representative of the stream network. However, because the monitored network tended to sample larger and higher order streams, they had lower median particle sizes, slopes, and proportions of some land uses like forestry (Table S2). Monitored sites also tended to be more likely classed as pastoral, low elevation and have either alluvial or volcanic acid geology than the whole network. These differences reflect past decisions by Regional Authorities of where water quality was changing and was poor^24^.

### Model performance

We considered a few approaches and models to predict median concentrations, standard deviations, and power in monitored and unmonitored sites (see Supplementary Information). Approximately the same proportion of sites (28%) had increasing or decreasing trends in contaminant concentrations (Table S3, Figs. S1-9). While we included trends in our model to predict concentration, we chose not to adjust trends for flow on the basis that flow is influenced by climate and would therefore require daily climate variation be predicted to predict future concentrations. Furthermore, the inclusion of flow as a co-variate tended to have little effect (< 10%) on standard deviations for most contaminants with the notable exception of turbidity (23.6%), which as a proxy for sediment, tends to be sensitive to high flows (Table S4, Fig S10). However, as flow data were only available for fewer than half of the sites, we did not include flow in our final models (see Supplementary Information). We recognise that not including flow the resulting models may underestimate variability in the data, especially for turbidity.

Linear models were developed to predict contaminant concentrations from a set of common site variables (Table S5) that used 13–26 localised or catchment-average terms (mean = 21) for biophysical conditions, and the results yielded coefficients of determination ranging from 0.37 for ammoniacal N (NH_4_-N) to 0.67 for total nitrogen (TN) (Table S6). There was no clear pattern in the number of localised versus catchment-average terms by contaminant.

Standard deviations were produced for each monitored site using a GAM. The GAM was able to account for seasonality over time and produced lower standard deviations than linear models for all parameters except clarity and turbidity (Fig. S11). The GAM-derived standard deviations were then used in models to predict the standard deviation in all unmonitored stream segments of 3rd Strahler order and greater using either a linear or random forest approach. The models used a mix of between 8 and 23 catchment variables (Table S7). Slope, geology, elevation, exotic forestry or intensive agriculture, and particle size were the most frequently used terms in the models. The coefficients of determination for models to predict standard deviation ranged from 0.30 to 0.67. The random forest models performed better (mean coefficient of determination = 0.51, mean squared error = 0.033) than the linear models (mean coefficient of determination = 0.40, mean squared error = 0.043) (Table S7) and hence chosen for the scenario analyses.

**Figure 3:**
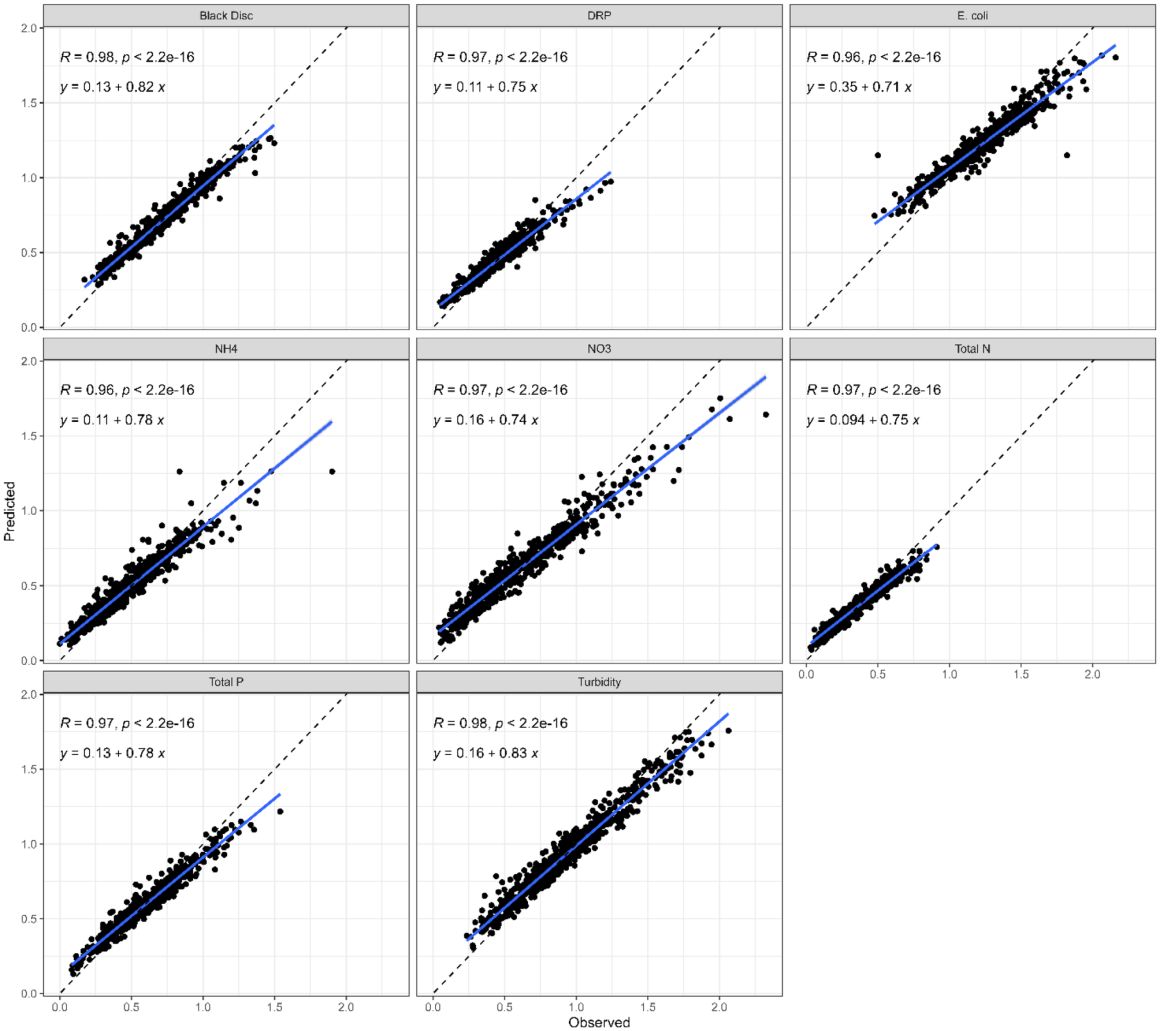
Plot of the predicted (via the random forest models) versus observed standard deviations for each contaminant.

**Figure 4:**
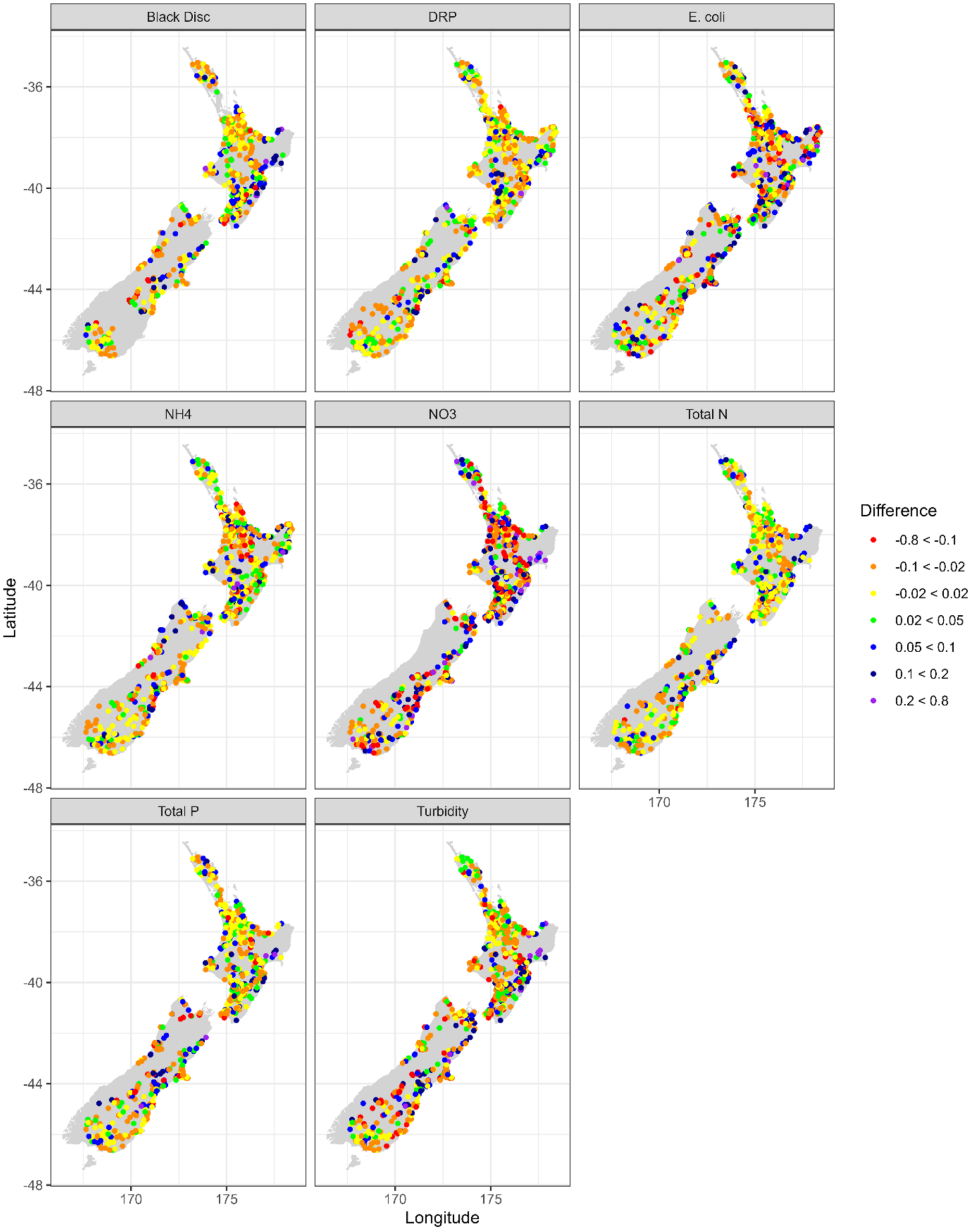
Map of the percentage residuals (log space: observed–predicted values, meaning that negative values are being over-predicted) for each site and contaminant.

We also plotted standard deviations from the random forest models against observed standard deviations for monitored sites to see if there was general agreement along the 1:1 line (Fig. 3). The output showed high agreement (*R*^*2*^ > 0.95, *P* < 0.001), but an under-prediction of standard deviations by 17–29%. When mapped there was no clear geospatial pattern to either over- or under-prediction of standard deviations, except for NO_3_-N which tended to be over-predicted in the central North Island (Fig. 4). Only the observed standard deviations were used in scenarios 1 and 2, but the plot of observed versus modelled data (Fig. 3) suggests there would have been only modest differences if the modelled data were used. Nevertheless, readers are advised that the consistent underprediction could lead to recommendations that too few samples are collected to detect a change in water quality. We therefore recommend that any new data be used to regularly reassess power.

### Costs

Mean costs from four (out of 16) Regional Authorities in New Zealand (Table S3) were used to assess monitoring costs associated with Scenario 1 (meeting threshold levels or maintaining the current state) and Scenario 2 (a 30% decrease in concentrations).

For Scenario 1, 14, 49, 247, 270, and 470 sites exceeded their respective threshold concentrations for NH_4_-N, NO_3_-N, clarity, dissolved reactive phosphorus (DRP), and *E. coli* by a median percentage of 46, 37, 41, 42, and 58%, respectively. Generally, sufficient power (i.e., ≥ 0.80) was available to calculate the number of samples and cost for > 95% of these sites. Monthly sampling did not meet the median frequency required to detect the level of change needed to reach the threshold for *E. coli* in 5 years (Table 1).

**Table 1:** Output of scenario 1 which lists the mean (and median in parentheses) number of samples per annum across monitored water quality sites (≥ 3rd order, n = 856) required to meet a minimum power of detection of ≥ 0.80 for water quality thresholds^1^ (or maintain current sampling where above the threshold) in either 5 or 20 years for each contaminant, along with the cost ($M NZD) per contaminant and sum and annualised cost. Total N, total P and turbidity assume regular monthly sampling is maintained for all sites. The costs for individual contaminants assume individual laboratory charges, Capex and Opex, and labour and mileage associated with the most frequently sampled contaminant at a site.

Contaminant/cost	Change within 5 years		Change within 20 years	
	Minimum number of samples	Annual cost	Minimum number of samples	Annual cost
Clarity	40 (12)	5.5	30 (12)	4.0
NO_3_-N	14 (12)	2.2	12 (12)	1.8
NH_4_-N	13 (12)	2.0	11 (12)	1.7
Total N	Na^2^	2.0	Na	1.6
Dissolved reactive P	30 (12)	4.8	19 (12)	3.1
Total P	Na	2.0	Na	1.6
Turbidity	Na	1.8	Na	1.5
*E. coli*	62 (26)	10.7	35 (12)	5.5
Sum assuming costs for minimum number of samples for each contaminant				
Total cost ($M NZD)		77		237
Annualised cost ($M NZD)		15.4		11.9

^1^Bottom lines are the threshold between C and D class waters in the NPS-FM^11^ listed as: the median clarity (by class of river) varying from 0.61 to 2.22 m; median concentration of 2.4 mg L^-1^ NO_3_-N, median concentration of 0.24 mg L^-1^ (at pH 8.0 and at 20 °C) for NH_4_-N; and the 95th percentile over 5 years at bathing sites for primary contact of 540 most probable number of *E. coli* 100 mL^-1^. The threshold for dissolved reactive P is 0.018 mg L^-1^, which we used as a proxy to indicate impact through periphyton growth^55^, above natural reference conditions, but is not listed as a bottom line in the policy.

^2^Na = not applicable as this contaminant has no bottom line or equivalent for rivers.

Annual costs in Scenario 1 were lower over 20 than five years ranging from $1.8–10.7 M in 5 years to $1.5–5.5 M in 20 years. The sum costs to monitor all eight contaminants were $77 M and $237 M over 5 and 20 years, but annual costs were $15.4 M and $11.9 M, respectively. This is approximately 5.3 and 4.1 times the current mean annual estimated cost of monitoring ($2.9 M). Note that both our estimates and the current cost of sampling only consider employment during sampling and data quality assurance steps. For example, if assuming only monthly sampling, current costs would increase by the ratio of full employment (e.g., 230 days after removing holidays and weekends) to sampling (12 days).

For Scenario 2 we calculated the mean and median number of samples per annum across monitored sites (≥ 3rd order; n = 856) needed to meet a minimum power of detection of 0.80 for an improvement target of 30% in either 5- or 20-years. After 5-years the median minimum number of samples ranged from 26 for ammoniacal-N (NH_4_-N), total N, and dissolved reactive P to 364 for *E. coli* and turbidity. Over a 20-year period the mean minimum number of samples was 12 for all contaminants except clarity and NO_3_-N which required fortnightly sampling and turbidity and *E. coli* which required weekly sampling (Table 2). If sampling only focused on one contaminant the annual cost ranged from $1.9 M for dissolved reactive P over 20-years to $62.3 M for *E. coli* over 5-years (Table 2). However, if only the minimum number of samples needed to detect change were collected for each contaminant, then the annual cost was $60.5 M for 5-years and $12.5 M for 20-years. Costs were greater than for scenario one owing to the greater number of sites requiring a change (i.e., some sites in scenario 1 already met their respective thresholds and were assumed to not require additional monitoring) and because we included total N, P and turbidity in scenario 2.

**Table 2:** Output of scenario 2 which lists the mean (and median in parentheses) number of samples per annum across monitored water quality sites (≥ 3rd order, n = 856) required to meet a minimum power of detection of ≥ 0.80 for a reduction target of 30% in either 5 or 20 years for each contaminant, along with the cost ($M NZD) per contaminant and sum and annualised cost. The costs for individual contaminants assume individual laboratory charges, Capex and Opex, and labour and mileage associated with the most frequently sampled contaminant at a site. The sum assumes costs (Capex, Opex, labour and analytical) are incurred for a minimum number of samples needed for all contaminants at each site.

Contaminant/cost	Change within 5 years		Change within 20 years	
	Minimum number of samples	Annual cost	Minimum number of samples	Annual cost
Clarity	96 (104)	13.1	22 (26)	3.0
NO_3_-N	94 (56)	14.5	22 (26)	3.4
NH_4_-N	42 (26)	6.6	14 (12)	2.3
Total N	29 (26)	4.8	12 (12)	2.0
Dissolved reactive P	30 (26)	4.8	12 (12)	1.9
Total P	66 (52)	10.8	17 (12)	2.9
Turbidity	234 (364)	34.5	40 (52)	6.0
*E. coli*	364 (364)	62.3	73 (52)	12.5
Sum assuming costs for minimum number of samples for each contaminant				
Total cost ($M NZD)		302.7		250.3
Annualised cost ($M NZD)		60.5		12.5

Using a national (random forest) model to predict standard deviation and power, we also applied the 30% reduction to unmonitored sites (viz. reaches) on all ≥ 4th order streams (n = 66,851). To detect change over 5-years the median number of samples per annum ranged from 26 for NH_4_-N, total N and dissolved reactive P to 364 for turbidity and *E. coli* (Table 3). To detect change over 20-years between 12 and 52 per annum were required (Table 3). This suggests that the current monitoring design (monthly) is only suitable to detect change over the relatively long-term, and only for some water quality measures.

**Table 3:** Mean and (median in parentheses) number of samples at sites in ≥ 4th order streams across the total river network (n = 66,851) required to meet (or unable to meet) a minimum power of detection of ≥ 0.80 for a reduction target of 30% in either 5 or 20 years.

Contaminant	5 years		20 years	
	Mean	Median	Mean	Median
Clarity	99	105	24	26
NO_3_-N	92	104	23	26
NH_4_-N	31	26	13	12
Total N	32	26	12	12
Dissolved reactive P	28	26	12	12
Total P	69	52	18	12
Turbidity	259	364	42	52
*E. coli*	364	364	68	52

## Discussion

### Limitations

Both the data and models produced come with caveats around their implementation. The user should be aware of these caveats, especially if using the model in a compliance setting that connects land use and management to water quality response. We will discuss more about the policy response in the next section; however, in the meantime the caveats can be classified into three aspects: 1) the spatial and temporal representativeness of the data and models, 2) the utility of the models to inform new sampling regimes, and 3) the accuracy of the models.

In filtering our data, we aimed to ensure that the dataset used for modelling was as representative of the national river network as possible. Despite large improvements in the representativeness of median values post filtering (Tables S1 and 2), Fig. 1 indicates that there are areas of New Zealand that are under-represented, such as the West Coast of the South Island, where additional data may improve spatial representativeness and model predictions. However, much of the West Coast is in permanent native forest and is not the target of policies aiming at improving water quality, so the need to monitor these sites must be balanced against the need to monitor sites that are exhibiting, or under threat of exhibiting, poor water quality.

We also filtered our data to include sites with as many observations as possible over a 15-year period. This helps ensure that changes in water quality concentrations are real. Recent work focusing on a subset (~ 10%) of monitored sites indicated that many of the trends in NO_3_-N concentrations for sites with < 10 years of data were caused by the Southern Oscillation Index and not land management^12^. This places emphasis on longer (e.g., 15–20-year) data records.

Our power calculations were done using monthly observations and are applied to percentage change of the mean value. Although, some work has shown that mean concentrations derived from annual or monthly data are just as variable as those collected days or hours apart^25^, most other studies show the opposite^26–28^. To test if our power calculations calculated on monthly observations would have been different if based on a data collected more frequently we obtained data from sites with observations for NO_3_-N (n = 9) and turbidity (n = 15) collected at 30-min intervals. These were the only high-frequency data and contaminants available and although they come from four Regional Authorities, we recognise that they may not represent the breadth of biophysical characteristics and contaminant responses possible across the country. After calculating standard deviations for both contaminants at different sampling frequencies, outputs were generally smaller for samples taken on a 30-min, daily or weekly interval compared to monthly sub-sampling (see Supplementary Information; Table S8). However, the higher standard deviations evident for monthly sampling avoids the risk of underestimating the number of samples required to detect change. More frequent samples in the short term will improve some applications, like estimating loads, because it will increase the chance of sampling infrequent high flows that dominate annual yields^27^. However, to detect changes it is more valuable to collect infrequent (viz. monthly) data over a long period than collect data more frequently over a short period. This is because effects may phase in and out or have a trend which would not be detected over short periods. Since about half the sites exhibited an increasing or decreasing trend (Table S3) for most of the contaminants (clarity showing the fewest trends), we argue that despite being sampled monthly, the 15-year period that our estimates are based off makes them as robust as possible.

A high accuracy of prediction is the usual aim of modelling. To better represent seasonality, we chose to estimate standard deviations over time with a GAM over a simple linear approach, only choosing a linear model where GAMs could not be produced (< 5% of sites). To predict standard deviation (and hence power) in unmonitored sites we used a random forest model. This approach has been used in other studies of water quality in New Zealand and generally accounts for a high amount of variation in the data (e.g., R^2^ > 0.60 for all contaminants^29,30^). However, we chose to not employ other machine learning techniques to maximise the amount of variation accounted for. This was deliberate as it may produce a higher statistical power that would increase the risk of underestimating the number of samples required to detect change. We considered our approach to be a good compromise between representing change at a site over time (via a GAM) and using those data in a random forest model that would capture an adequate level of variation^31^ but not over fit the data.

### Implications for policy

The recommended frequency of sampling, and hence implications for cost, depends on the objective. Jordan and Cassidy^32^ outline a range of sampling options that vary from “business as usual” routine grab sampling to mobile real-time monitoring systems. High frequency sampling, either *in-situ* or remote, is useful for distinguishing catchment processes^33^, getting better estimates of flux^34^, and could be used for allocation if widely used to distinguish processes across a catchment^35^. However less frequent sampling over a longer period can be sufficient to account for state and trends. Our analysis examined routine monthly sampling that over a long period is useful for state and trend analyses, and it generally assumed to be cost effective, if located in the right place^15^.

Much research has examined where sampling should occur based on a mix of semi-quantitative risk assessments^36^ and quantitative machine learning techniques that optimise solutions towards an objective^15,37^. However, recommendations for monitoring need to link to policy objectives that in turn link to on farm actions to prevent waterway contamination at appropriate spatial and temporal scales. For instance, catchment or farm modelling can identify small areas that contribute most of the contaminant load, commonly termed critical source areas, which can help prioritised cost-effectiveness to mitigate contaminant loss^38^. The greatest chance of detecting the effect of those actions is closer to where they are implemented^20,39^. However, most critical source areas exhibit episodic losses in response to rainfall implying that routine regular sampling may not adequately capture periods of high concentrations or loads and that a more frequent and expensive sampling regime is needed. Clearly a mix of sites is required where some are used to detect long-term trends and other used to confirm the effectiveness of actions in a time and cost-effective manner but to reduce costs their location could be informed by our national model to direct monitoring towards more sensitive sites likely to have high power and exhibit change quickly. These action-focused sites could be used to confirm catchment- or sub-catchment scale effectiveness of management interventions, which may be used in the future to guide where actions should occur elsewhere.

We tested New Zealand policy that aimed to start making improvements within five years and bring waterways to a healthy state within a generation (viz. 20-years) by framing this within current water quality monitoring efforts^22^. Our first assumption (and Scenario) was that this would require some change in sampling for current water quality sites to detect whether “bottom line” water quality thresholds established by national legislation are met. The Parliamentary Commissioner for the Environment expressed some scepticism that current networks would be able to monitor change effectively and that government resourcing may need to increase to do so^40,41^. We can confirm that to detect whether thresholds are reached for the sub-set of contaminants we tested, investment in monitoring would have to increase by 4 to 5 times current levels. However, this cost is likely to increase further considering that the current National Objective Framework within New Zealand’s NPS-FM^11^ outlines 22 water quality and ecological attributes applicable to rivers and lakes that require consideration, of which about half are directly linked to the contaminants we tested. Communities may seek better water quality than “bottom line” thresholds, which will mean that improvement will need to be detected at more sites (e.g., as evidenced by the blanket decrease and increased cost in Scenario 2), increasing sampling effort and cost. Clearly, without substantial investment, this will not be achievable. Some technological fixes, such as remote sensing and high-frequency monitoring apparatuses could, in the future, bring the cost down. However, a more sensible approach may be to rationalise the current network, focusing investment on increasing sampling of fewer sites, identifying a better mix of sites that may quickly respond to actions to improve water quality and sites to show long-term trends^39^.

## Methods

We utilised a checked and filtered dataset of stream and river concentrations and discharge to estimate standard deviations of key contaminants. We then use these data with a set of predictors to model concentrations and standard deviations nationally. The outputs from these predictions were used to estimate power and produce an interactive map from which a user can select a combination of two factors to predict a third. The factors offered were sampling duration, sampling density and the likelihood of detecting a percentage change from a baseline trajectory. We used these data and the map to test two scenarios that assess the ability to detect change and the associated cost in 5- and 20-years. The process of filtering and modelling of data into the interactive map is outlined in Fig. 2.

### Dataset

We obtained site-specific contaminant concentrations from New Zealand’s 16 Regional Authorities (Authorities) via the Land, Air, Water, Aotearoa website (www.lawa.org.nz), and from the National Institute of Water and Atmospheric Research’s (NIWA) National River Water Quality Network (NRWQN). The contaminants included in our analysis were visual clarity (clarity), ammoniacal nitrogen (NH_4_-N), nitrate nitrogen (NO_3_-N); total N; dissolved reactive phosphorus; total phosphorus (total P); turbidity, and the faecal indicator bacterium *Escherichia coli* sp. (*E. coli*). Note that while visual clarity is not a contaminant itself, it is, like turbidity a good indicator of contamination by sediment or organic matter inputs.

As of 2022, Authorities and NIWA sample and maintain 985 sites. The sampling and maintenance of 42 (out of 77) of the NRWQN sites have transferred to Authorities in the last five years. Authority sites have been sampled since 1975 and the NRMN and NRWQN since 1989. A description of the sites, methods used, and quality of the data are available elsewhere^42–45^.

### Data filtering

Sampling in the NRWQN (monthly) and the analysis of water samples have been constant since their inception^46^. However, sampling intervals, analytical techniques and reporting conventions have varied amongst the Authorities. As outlined in previous work^18^ we used a multi-step process to check for data stored in incorrect units and impute replacement values for censored data. We chose data that were measured using consistent methods: alkaline persulphate digestion of unfiltered samples prior to making total N and total P measurements, a most probable number method for *E. coli*, horizontal sighting range of black disc for visual clarity, and colorimetry on samples for the measurement of dissolved reactive P. We considered NO_3_-N measurements made with ion chromatography, cadmium reduction, azo dye colorimetry or optical sensor to be comparable^47^. We excluded all other methods for clarity, *E. coli*, NH_4_-N, NO_3_-N, dissolved reactive P, total N, and total P from our database.

We restricted our database to samples taken between 1990 and 2019. This period allowed for the greatest consistency in analytical methods and reporting. We chose 15 years as a period which will account for trends owing to land use or climatic variation^48,49^. We also considered the full database to be unrepresentative of the national stream network, sampling far fewer smaller order streams. This reduced the number of sites for which statistical power could be calculated to 856. However, sampling was variable for some sites. To maximise the chance of detecting trends in a national model to predict power in unmonitored sites we removed a further 86 sites that had < 40 samples over ≥ 5 years of data.

### Modelling concentrations and power

**Figure 5:**
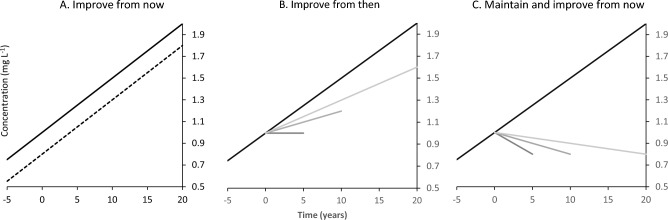
Graphical interpretation of a 20% improvement from now (**A**) or at five, 10 or 20 years in the future (**B**), or with the expectation that improvements are achieved relative to now, reached and maintained in five, 10 or 20 years in the future (**C**).

Following a preliminary analysis to help choose an appropriate set of models (see Supplementary Information), we used the following steps to predict concentrations (for reaches with monitoring sites) and standard deviation (for all reaches, e.g., both monitored and unmonitored) from which the power could be calculated for chosen sampling frequencies (Fig. 2):

1. For reaches with monitoring sites containing sufficient data, we fitted a linear model to predict the concentration and trend of the log of contaminant concentrations over the period of the observations (see Table S4 and Figs. S1-9 for the number of sites with a significant trend). We log-transformed data to normalise their skew and used these data for all models. These models were used to predict the concentration at a point in time, which must be set before a percentage decrease in contaminant concentrations can be considered. There were insufficient data and evidence to justify adjusting concentrations for flow (see Supplementary Information and Table S4 and Fig. S10). We then modelled the standard deviation with time for each site using a linear model (Eq 1: concentration = intercept + slope × time) and a GAM using the mgcv package in R^51^, but only used the standard deviations outputs from the GAMs in subsequent power calculations as they better account for seasonality in the time series than the linear approach and generated lower standard deviations (see Supplementary Information for a comparison of the linear and GAM outputs, Fig. S11).
2. For monitored sites power predictions were made for a range of percentage reductions in predicted concentrations for the 1^st^ of January 2022 (year 0 in Fig. 5). The power was estimated by interpolating a linear model (Eq 1) for the proposed, decreasing, trend (e.g., the five years before 2022 in Fig. 5) and adding a random component as the predicted standard deviation via the GAM outputs. A linear model was then fitted, and the slope tested for significance, i.e., an interaction term between time after 0 and the slope would be significant and negative. This was repeated 10,000 times and the proportion of significant negative (*P* < 0.05) slopes was used as the estimate of power.
3. For all reaches with monitoring sites, we collected a range of attributes for the reach outlet and upstream catchment likely to influence concentration and standard deviation of the trends (Supplementary Table S5). We combined these attributes with GAM predictions of standard deviation for monitored sites and produced a set of linear and random forest models for each contaminant to predict standard deviation for unmonitored (and monitored) sites. However, like step 1 we only used the random forest models for power predictions at unmonitored sites owing to their better fit than the linear models. The random forest model employed the randomForest package in R^50^ with the default parameters, 500 trees and 10 variables tried at each split. The model was fitted to a training data set with 75% of the observations and tested with the remainder, if the fit was adequate the model was refitted to all the data and variables selected by inspection of the variable importance graph. The final models excluded terms that were not significant (*P* > 0.05) leaving a sensible model that predicted contaminant concentrations and standard deviations well (determined via the Mean Square Error and Coefficient of Determination; we also produced the Akaike Information Criterion^52^ for the linear models but note that a true AIC is not possible for the random forest models). The final linear and random forest models are shown in Supplementary Tables S6 and S7. For unmonitored sites, power calculations were made using the standard deviations estimated from the random forest models. We did not predict trends for unmonitored sites.

Predictions of power were made for any interval but to facilitate faster processing at the catchment scale in the interactive map were calculated at 5% intervals for the following combinations: sampling intervals of daily, twice weekly, weekly, fortnightly, monthly, and quarterly; sampling periods of 2, 5, 10, 20, and 30 years into the future; and reductions of 5, 10, 15, 20, 25, 30, 40, and 50%.

### Interactive map

The interactive map application (https://www.monitoringfreshwater.co.nz/ and Fig. 2) allows the user to explore power by first selecting a catchment in New Zealand of ≥ 3rd order according to the River Environment Classification (REC, v2.5)^53^ that flows to the coast, stream or river reach. The user can select all reaches in the catchment of a specific set of reaches (viz. points). The user chooses a contaminant and then is asked to define the expected relative water quality change and then selects (1) the sampling period, (2) the number of samples, and (3) the percentage reduction in contaminant concentration. Once options are chosen either the observed or predicted power is output for specific points with observations (monitoring sites) and predictions for unmonitored stream reaches. Facility has also been built into the map to upload a polygon layer of the catchment that represents potential reductions to receiving streams and rivers based on likely farm types and mitigation actions^23,54^.

The map was developed in the Python programming language using the Dash web application framework (https://dash.plotly.com/). The application routes the reductions downstream using weighted loads of the associated contaminant derived from land use-based losses and flows from the REC v2.5^23,53^.

We restricted the map’s outputs by removing those sites that were in the conservation estate and first or second order streams. Water quality in the conservation estate is good and unlikely to be intensified^24^. We removed small (order 1 and 2) streams as they are poorly represented in the database (< 15% of sites) compared to the large proportion (> 65%) of small streams in the national network.

### Scenario testing

We defined improvement as either an ‘improvement’ on the current trajectory for water quality, or as defined in policy^11^ as ‘maintain and improve’ from the current concentration. We accounted for trends in the modelling of concentrations and standard deviations. Predictions of an appropriate sampling regime included any positive or negative trend in the data over the timeframe chosen.

We outline three cases of how the terms ‘improvement’ and ‘maintain and improve’ could be interpreted setting an example target of a 20% decrease and a current median NO_3_-N concentration of 1 mg L^-1^ (Fig. 5).

A. Improve (from now, year 0) aims to achieve an instantaneous 20% decrease (i.e., to 0.8 mg L^-1^) but increasing at the same rate thereafter. We did not include this scenario in our analysis as it is unlikely to occur except where there is no trend, in which case it would default to scenario C.
B. Improve (from then) aims to decrease concentrations at a point in the future. If we assume a positive trend in annual median NO_3_-N concentration (e.g., in the 5 years prior to year 0) equivalent to 5% per annum was detected and maintained at the same magnitude, then in 10 years the concentration will be 1.5 mg L^-1^. A user choosing to see a 10% improvement in 10 years would be sampling to detect a concentration of 1.2 mg L^-1^.
C. Maintain and improve aims to reach the 20% decrease relative to now (year 0 = 1.0 mg L^-1^) but at a point in the future. For example, at year 10 with the concentration now at 1.5 mg L^-1^ a decrease of 0.7 mg L^-1^ is required to reach a concentration of 0.8 mg L^-1^, effectively a 47% decrease at year 10.

In this paper we examine case C (maintain and improve relative to now as a one-sided hypothesis test that the decrease in slope is significant) setting a target in monitored ≥ 3rd order rivers (n = 856) in 5- and 20-years from the first of January 2022 in two scenarios. We chose ≥ 3rd order rivers to reflect the minimum scale at which the NPS-FM is likely to apply and because this scale represents a compromise between being small enough to detect changes in land use and land use practices early and large enough that regional authorities would not have to monitor too many sites. The two targets were:

1. Scenario 1. Meeting threshold values in the NPS-FM^11^ relating to bottom lines for clarity, toxicity for NH_4_-N and NO_3_-N, and primary contact in bathing areas for *E. coli*, and a threshold between C and D class waters that relates to an enhanced impact on water quality through periphyton growth^55^ for dissolved reactive P (see Table 1).
2. A 30% decrease in medians at all monitored sites.

Because our work can be used to detect change at new sites, we also estimated the median number of samples required to meet a 30% reduction in 5 and 20 years in all ≥ 4th order rivers across the network (all stream order; n = 66,851). We chose 4th order streams because their biophysical characteristics (e.g., slope, land use) are more consistent than 3rd order streams.

### Cost estimates

Estimates of costs associated with the collection of monthly water quality data were collated from four Regional Authorities and three commercial water quality analytical laboratories in New Zealand. Cost estimates were obtained for staff time—including preparation time, travel time to get to site sample/measurement collection and data entry and QA/QC procedures, and analytical costs—including equipment purchase for in-field measurements and commercial analytical costs. Costs associated with the development or maintenance of databases or data systems were excluded. We derived an average travel time and mileage cost per site and per sampling event based on the total distance covered and total number of sites in each authorities’ network. Further information about costs is given in the Supplementary Information (and Table S9).

### Supplementary Information

Supplementary Information.

## Data Availability

Filtered input data, power and cost calculations can be found at: https://figshare.com/s/8e31cbb1ff9565023487
