# Supplementary material for: Monitoring to detect changes in water quality to meet policy objectives
Source: Sci Rep. 2024 Jan 22;14:1914. doi: 10.1038/s41598-024-52512-7 (PMC10803785; doi:10.1038/s41598-024-52512-7)
Supplement: Supplementary file 1 — Supplementary Information. [file 41598_2024_52512_MOESM1_ESM.docx]

# Supplementary Information

## Model variables

**Table S1**. Catchment-specific continuous and categorical variables (excluding date) included in the assessment of the models (Table S6-7) used to predict the concentration for each contaminant. Note that all predictors were used in models to predict standard deviations while local versions (within 50 m of the reach terminus) of the same variables (denoted by the prefix loc_ in the code).

| Variable (abbrev name) | Description and unit | Source |
| --- | --- | --- |
| lawa_or_NRWQN_ID | Site ID | ^1^ |
| HydroID | One of the ID variables in the REC dataset; each unique stream segment has one. | ^2^ |
| nzsegment | Ibid, just a different ID from the REC -- I believe each segment has a unique HydroID and a Unique reach number within the New Zealand hydrological network of version 2 of the River Environment Classification (REC). | ^2^ |
| nzreach_rec1 | Unique reach number within version 1 of the REC | ^3^,^2^ |
| StreamOrder | Strahler stream order | ^2^ |
| sinuosity | Actual distance divided by the straight-line distance giving the degree of curvature of the stream | ^2^ |
| us_ind | Upstream (catchment) induration or hardness value. ordinal scale | ^2^ |
| us_elev | Upstream (catchment) mean elevation above sea level of the watershed or basin. m | ^2^ |
| us_slope | Upstream (catchment) mean slope of the watershed or basin in degrees. Degrees | ^2^ |
| us_tmin | Upstream (catchment) mean minimum wintertime air temperature. deg C x 10 | ^2^ |
| us_june | Upstream (catchment) June solar radiation. W/m2 | ^2^ |
| us_penpet | Upstream (catchment) penman potential evaporation measurement. mm | ^2^ |
| us_rnvar | Upstream (catchment) Coefficient of variation of annual catchment rainfall. mm | ^2^ |
| us_rd10 | Upstream (catchment) Catchment rain days (greater than 10mm/month). mean # days/mo | ^2^ |
| us_rd20 | Upstream (catchment) "rd20","rd25","rd100" are similar to above, except for the threshold rain. | ^2^ |
| us_rd25 | Upstream (catchment) "rd20","rd25","rd100" are like above, except for the threshold rain. | ^2^ |
| us_rd100 | Upstream (catchment) "rd20","rd25","rd100" are like above, except for the threshold rain. | ^2^ |
| us_phos | Upstream (catchment) "phos" - Catchment average of phosphorous. ordinal scale. | ^2^ |
| us_psize | Upstream (catchment) "psize" - Catchment average of particle size. ordinal scale. | ^2^ |
| us_pet | Upstream (catchment) "pet" - Annual potential evapotranspiration of catchment. mm | ^2^ |
| us_mat | Upstream (catchment) "mat" - mean maximum (?) air temperature. deg C x 10. | ^2^ |
| us_decs | Upstream (catchment) "decs" - December catchment solar radiation. W/m2. | ^2^ |
| us_twarm | Upstream (catchment) "twarm" - Average within section mean January air temperature. deg C x10 | ^2^ |
| us_catarea | Upstream (catchment) "catarea" - catchment area. m2 | ^2^ |
| CLIMATE | Climate class within the REC | ^3^ |
| SRC_OF_FLW | Source of Flow within the REC | ^3^ |
| GEOLOGY | Geology within the REC | ^3^ |
| LANDCOVER | Landcover class within the REC | ^3^ |
| NET_POSN | Net position class within the REC | ^3^ |
| VLY_LNDFRM | Valley landform class within the REC | ^3^ |
| SPRING | Indicator for whether the segment is a 'spring' | ^3^ |
| lcdb_v3_usBare | See Table 2-2 from Whitehead ^4^, units are in % of catchment area, based on Manaaki Whenua Landcare Research ^5^ | ^4,5^ |
| lcdb_v3_usExoticForest | See Table 2-2 from Whitehead ^4^, units are in % of catchment area, based on Manaaki Whenua Landcare Research ^5^ | ^4,5^ |
| lcdb_v3_usIntensiveAg | See Table 2-2 from Whitehead ^4^, units are in % of catchment area, based on Manaaki Whenua Landcare Research ^5^ | ^4,5^ |
| lcdb_v3_usNativeForest | See Table 2-2 from Whitehead ^4^, units are in % of catchment area, based on Manaaki Whenua Landcare Research ^5^ | ^4,5^ |
| lcdb_v3_usPastoralLight | See Table 2-2 from Whitehead ^4^, units are in % of catchment area, based on Manaaki Whenua Landcare Research ^5^ | ^4,5^ |
| lcdb_v3_usScrub | See Table 2-2 from Whitehead ^4^, units are in % of catchment area, based on Manaaki Whenua Landcare Research ^5^ | ^4,5^ |
| lcdb_v3_usUrban | See Table 2-2 from Whitehead ^4^, units are in % of catchment area, based on Manaaki Whenua Landcare Research ^5^ | ^4,5^ |
| lcdb_v3_usWetland | See Table 2-2 from Whitehead ^4^, units are in % of catchment area, based on Manaaki Whenua Landcare Research ^5^ | ^4,5^ |

## Representativeness

**Table S2**. Median values for the unfiltered and filtered monitored sites and all reaches on the river network. Difference indicates whether there is a significant difference (*P*<0.05; Mann-Whitney test) between the monitored sites and all reaches.

| Parameter | Monitored sites_unfiltered_ | All sites_unfiltered_ | Difference_unfiltered_ | Monitored sites_filtered_ | All sites_filtered_ | Difference_filtered_ |
| --- | --- | --- | --- | --- | --- | --- |
| Stream order | 4 | 1 | Yes | 5 | 3 | Yes |
| PET (mm yr^-1^) | 1004 | 927 | Yes | 979 | 966 | No |
| Elevation (m) | 314 | 453 | Yes | 340 | 381 | No |
| Slope (degrees) | 11 | 4 | Yes | 11 | 4 | Yes |
| Tmin (^o^C) | 17 | 7 | Yes | 17 | 15 | No |
| Hardness | 2.9 | 3.6 | Yes | 3.0 | 3.2 | No |
| Phosphorus | 2.2 | 2.5 | Yes | 2.1 | 2.3 | No |
| Particle size | 2.8 | 3.8 | Yes | 2.9 | 3.3 | Yes |
| Area (km^2^) | 88 | 1 | yes | 120 | 20 | Yes |
| Intensive Ag (%) | 41 | 0.3 | Yes | 40.3 | 35.0 | No |
| Low pasture (%) | 0.2 | <0.1 | Yes | 0.4 | 0.6 | No |
| Native forest (%) | 7.2 | 1.6 | Yes | 9.8 | 4.6 | Yes |
| Exotic forest (%) | 2.9 | <0.1 | Yes | 3.3 | 1.9 | Yes |
| Scrub (%) | 5.5 | 1.1 | Yes | 5.8 | 4.8 | No |
| Wetland (%) | <0.1 | <0.1 | Yes | <0.1 | <0.1 | Yes |
| Bare (%) | <0.1 | <0.1 | Yes | <0.1 | <0.1 | Yes |
| Urban (%) | <0.1 | <0.1 | Yes | <0.1 | <0.1 | Yes |

**Table S3**. Proportions of unfiltered and filtered monitored sites and all reaches on the river network within each level of the River Environment Classification^2^. Difference indicates whether there is a significant difference (*P*<0.05; hypothesis test) between the monitored sites and all reaches.

| River Environment Class | Monitored sites_unfiltered_ | All sites_unfiltered_ | Difference_unfiltered_ | Monitored sites_filtered_ | All sites_filtered_ | Difference_filtered_ |
| --- | --- | --- | --- | --- | --- | --- |
| Position |  |  |  |  |  |  |
| HO, High order | 47% | 6% | yes | 56% | 23% | yes |
| LO, Low order | 15% | 74% | no | 0% | 0% | no |
| MO, Middle order | 38% | 20% | yes | 44% | 77% | no |
| Landform |  |  |  |  |  |  |
| HG, High-gradient | 3% | 53% | no | 1% | 20% | no |
| LG, Low-gradient | 89% | 35% | yes | 92% | 65% | yes |
| MG, Medium-gradient | 7% | 12% | no | 7% | 15% | no |
| Landcover |  |  |  |  |  |  |
| B, Bare | 1% | 6% | no | 1% | 8% | no |
| EF, Exotic forest | 3% | 5% | no | 3% | 3% | no |
| IF, Indigenous forest | 16% | 24% | no | 17% | 26% | no |
| P, Pastoral | 66% | 42% | yes | 67% | 42% | yes |
| S, Scrub | 3% | 5% | no | 2% | 3% | no |
| T, Tussock | 5% | 16% | no | 6% | 17% | no |
| U, Urban | 7% | 1% | yes | 4% | 0% | no |
| Geology |  |  |  |  |  |  |
| Al, Alluvium | 21% | 11% | yes | 16% | 8% | yes |
| HS, Hard sedimentary | 29% | 40% | no | 32% | 44% | no |
| M, Miscellaneous | 6% | 5% | no | 6% | 3% | no |
| Pl, Plutonics | 1% | 6% | no | 1% | 7% | no |
| SS, Soft sedimentary | 20% | 21% | no | 20% | 21% | no |
| VA, Volcanic acid | 22% | 16% | yes | 24% | 17% | yes |
| VB, Volcanic basic | 2% | 1% | no | 1% | 1% | no |
| Source of flow |  |  |  |  |  |  |
| GM, Glacial-mountain | 1% | 3% | no | 1% | 5% | no |
| H, Hill | 29% | 34% | no | 33% | 35% | no |
| L, Low-elevation | 63% | 44% | yes | 58% | 36% | yes |
| Lk, Lake | 4% | 2% | no | 5% | 5% | no |
| M, Mountain | 3% | 17% | no | 3% | 17% | no |
| Climate |  |  |  |  |  |  |
| CD, Cool dry | 26% | 21% | no | 24% | 18% | no |
| CW, Cool wet | 37% | 32% | no | 40% | 36% | no |
| CX, Cool extremely wet | 8% | 24% | no | 9% | 26% | no |
| WD, Warm dry | 8% | 5% | no | 5% | 3% | no |
| WW, Warm wet | 20% | 17% | no | 20% | 15% | no |
| WX, Warm extremely wet | 2% | 1% | no | 2% | 1% | no |

## Selecting a model to predict concentration and standard deviations.

We considered two approaches to use site data for trends in concentrations and standard deviations to predict concentrations and standard deviations for eligible reaches nationally – a simple linear approach and a more complex machine learning random forest method. Both produced standard deviations that were used to estimate power and inform sampling schema. However, while the random forest model accounts for more variation, the lower standard deviations, compared to a linear approach, results in estimates of statistical power and a sampling regime that risks underestimating the number of samples required to detect change. Furthermore, many of the variables in the random forest model are poorly predicted at a fine scale (e.g., daily discharge or precipitation) which may impair predictions when features like a changing climate are interacting. Hence, we chose a linear model that accounts for less variation but boosts the standard deviation and the required number of samples. However, as many of the contaminants vary with season and / or flow, and users may wish to select a sampling scheme that is less than a year, we included smoothers, converting the model to a GAM to account for effects such as season (Mar-May, Jun-Aug, Sep-Nov, Dec-Feb) on model performance.

### Advice for adjusting trends for climate change.

Previous work has revealed that climate has a large effect on water quality trends in New Zealand rivers^6^. This result was discovered using the NRWQN 30-year dataset. Most of our sites have data for up to 15 years. We cannot discount the effect of climate variation on trends and predicting their response will be subject to additional modelling and prediction of climatic variation. However, if a site has long term data, power calculations we included a facility within the interactive map to upload the old data and compared it to the existing dataset. If a material change in trend analysis is found, advice is given to take a cautious approach to sampling.

### Accounting for trends

**Table S4**. Number of sites for each contaminant showing a significant (P<0.05) increasing or decreasing trend in concentration.

| Contaminant | Number of sites increasing | Number of sites decreasing | Number of sites in which the change estimate is not statistically significant |
| --- | --- | --- | --- |
| NO_3_-N | 169 | 105 | 608 |
| NH_4_-N | 196 | 245 | 441 |
| TN | 214 | 81 | 609 |
| DRP | 144 | 328 | 426 |
| TP | 89 | 237 | 571 |
| E. coli | 151 | 85 | 670 |
| Turbidity | 153 | 91 | 618 |
| pH | 221 | 155 | 448 |
| Clarity | 86 | 64 | 531 |


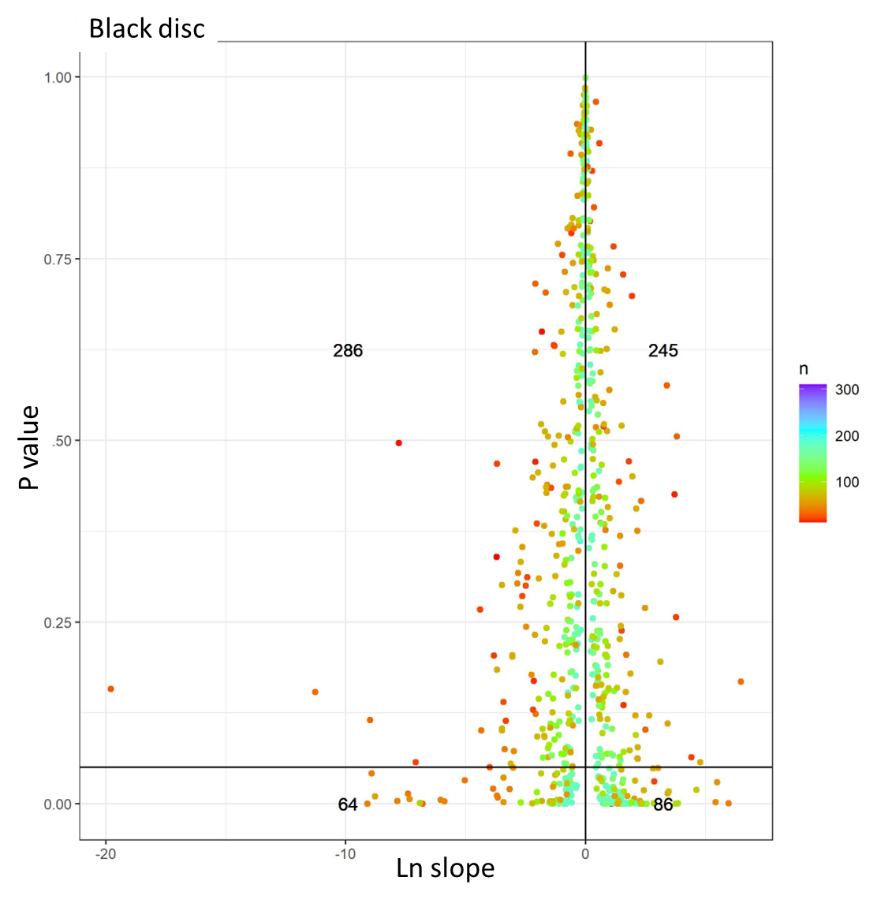


**Fig S1**. Count of sites with either increasing (right of vertical line) or decreasing (left of vertical line) trends in clarity (black disc) (log scale) that are significant (below horizontal line) or not (above horizontal line). N refers to the number of samples at each sampling point.


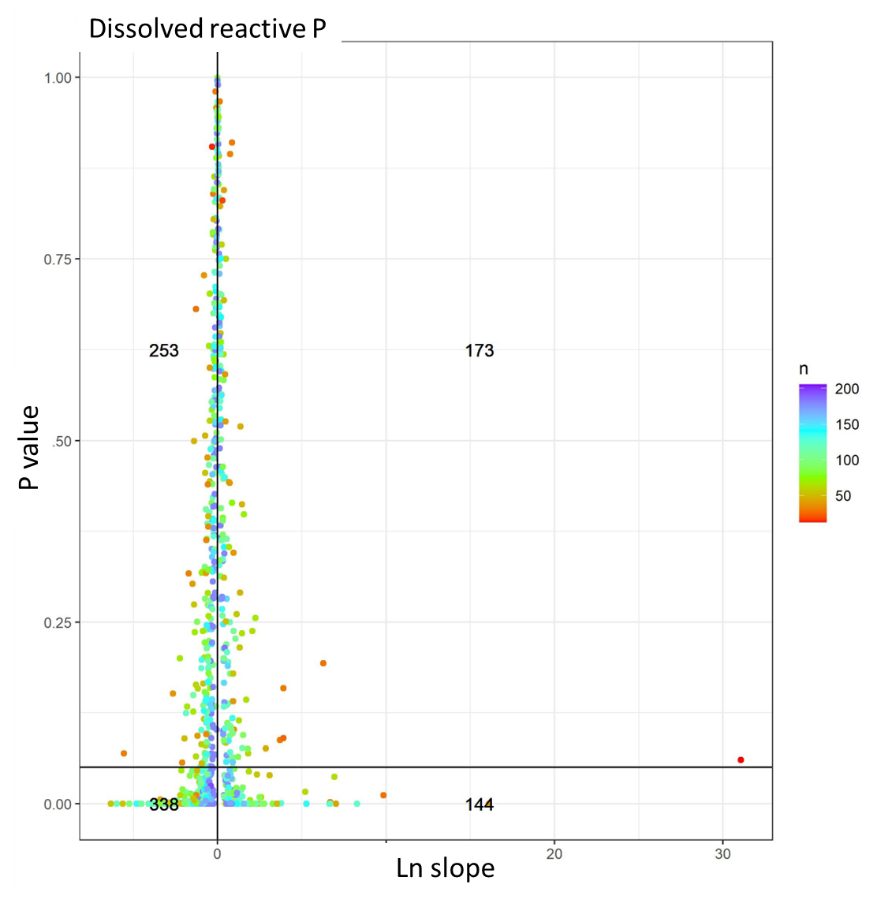


**Fig S2**. Count of sites with either increasing (right of vertical line) or decreasing (left of vertical line) trends in dissolved reactive phosphorus (log scale) that are significant (below horizontal line) or not (above horizontal line). N refers to the number of samples at each sampling point.


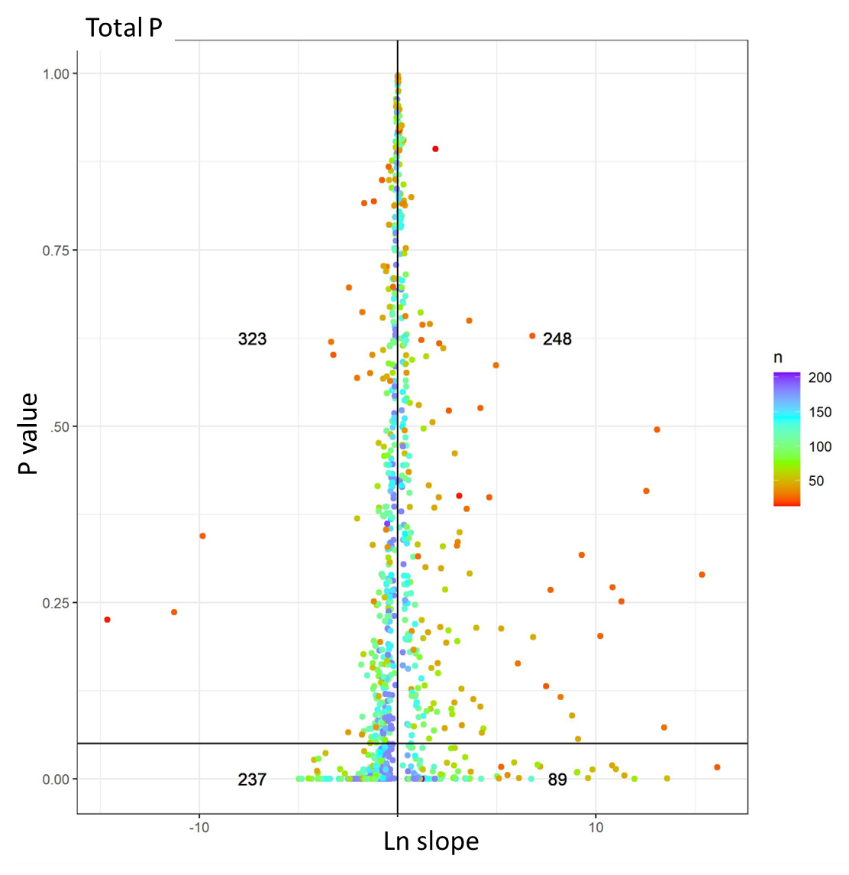


**Fig S3**. Count of sites with either increasing (right of vertical line) or decreasing (left of vertical line) trends in total phosphorus (log scale) that are significant (below horizontal line) or not (above horizontal line). N refers to the number of samples at each sampling point.


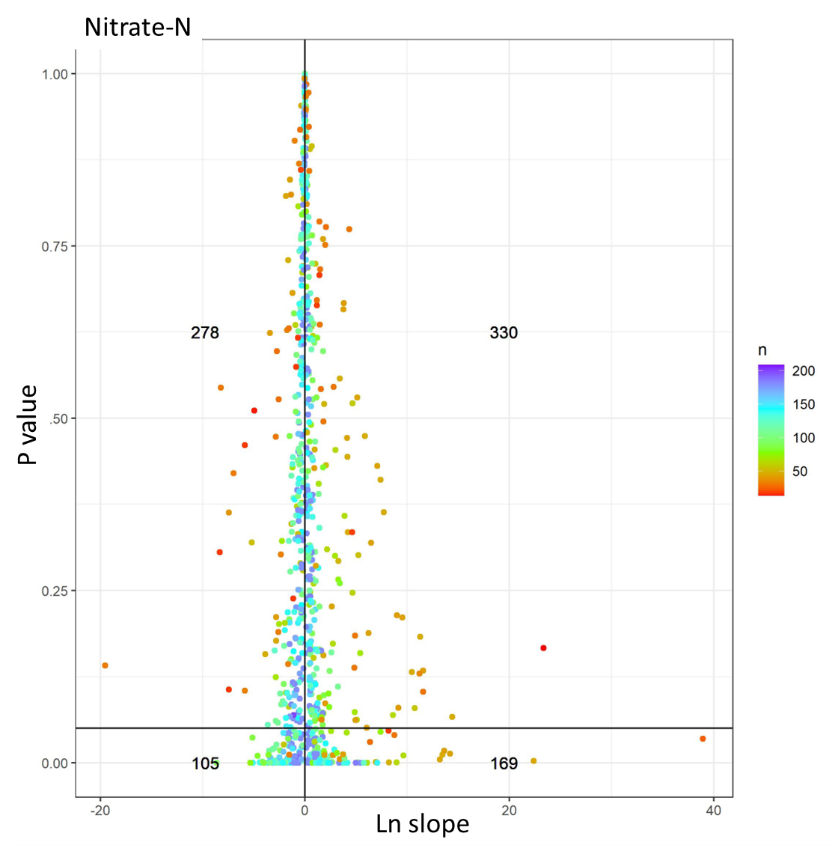


**Fig S4**. Count of sites with either increasing (right of vertical line) or decreasing (left of vertical line) trends in nitrate-nitrogen (log scale) that are significant (below horizontal line) or not (above horizontal line). N refers to the number of samples at each sampling point.


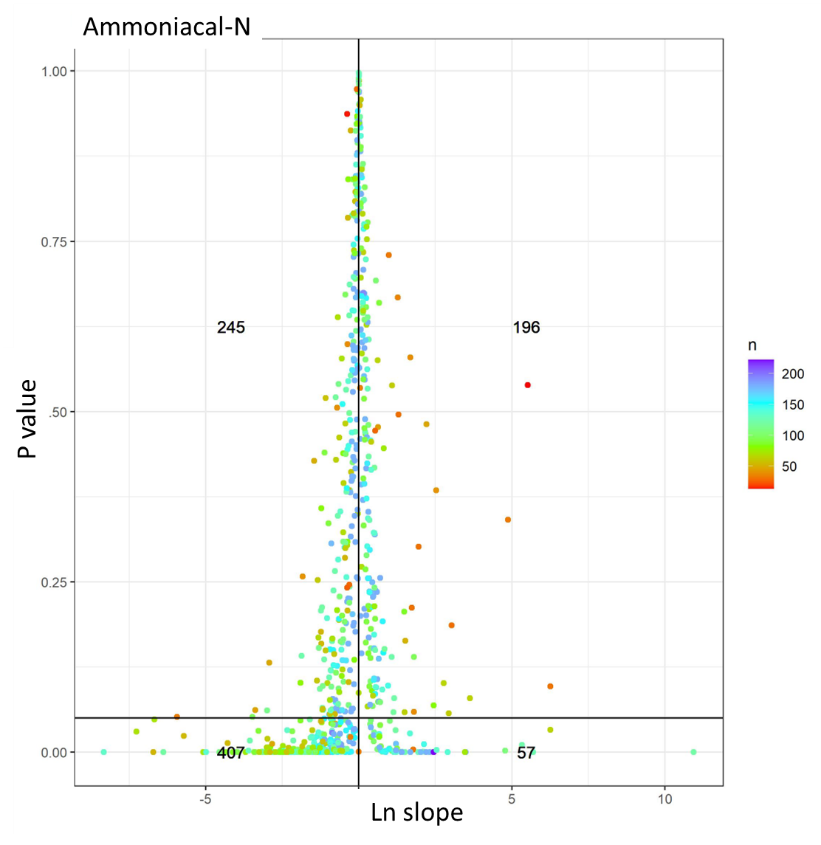


**Fig S5**. Count of sites with either increasing (right of vertical line) or decreasing (left of vertical line) trends in ammoniacal-nitrogen (log scale) that are significant (below horizontal line) or not (above horizontal line). N refers to the number of samples at each sampling point.


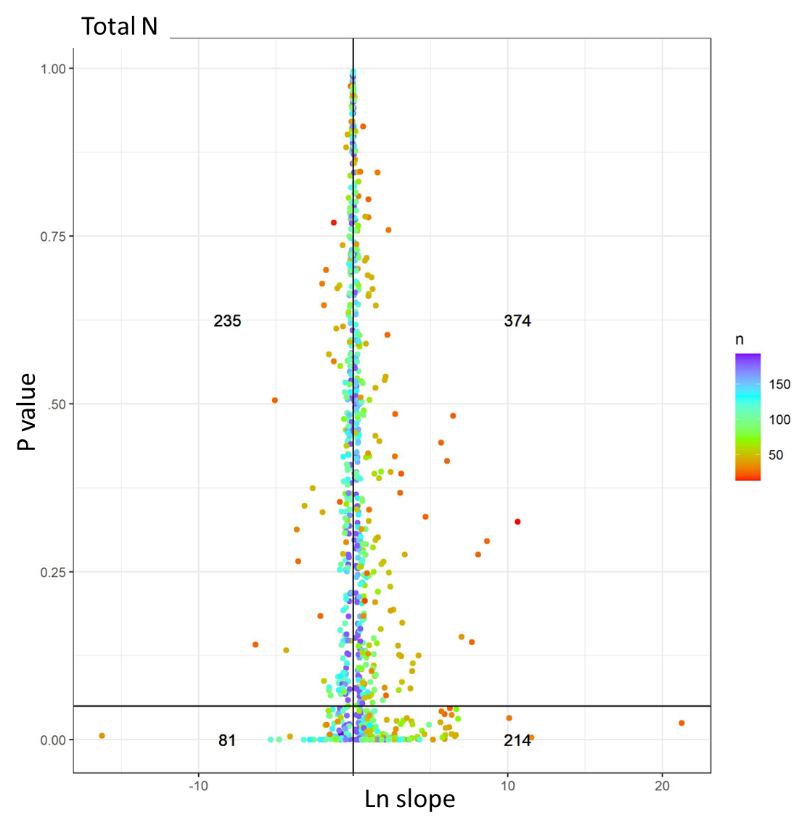


**Fig S6**. Count of sites with either increasing (right of vertical line) or decreasing (left of vertical line) trends in total nitrogen (log scale) that are significant (below horizontal line) or not (above horizontal line). N refers to the number of samples at each sampling point.


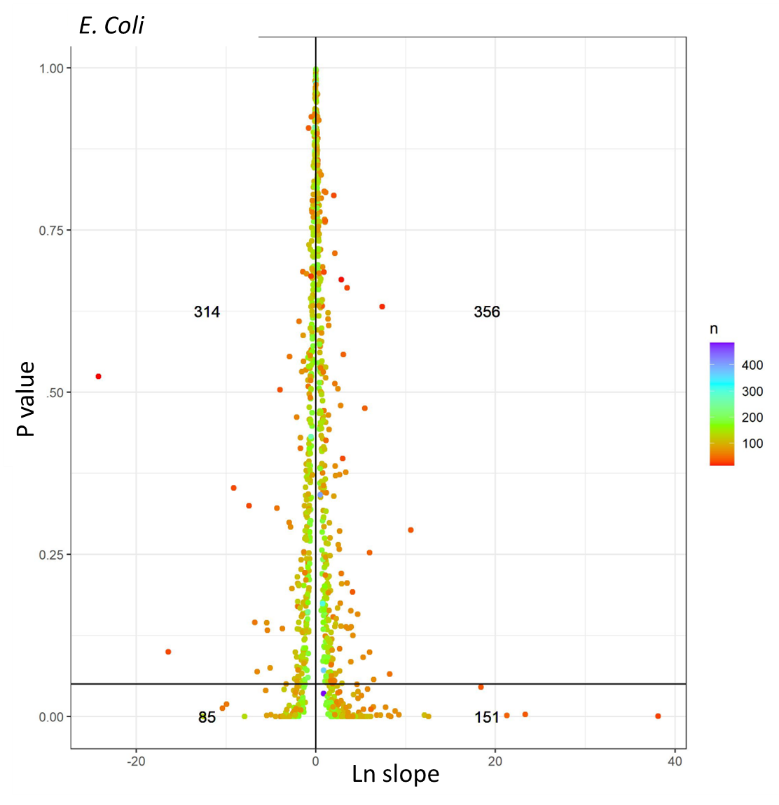


**Fig S7**. Count of sites with either increasing (right of vertical line) or decreasing (left of vertical line) trends in *Escherichia coli* (log scale) that are significant (below horizontal line) or not (above horizontal line). N refers to the number of samples at each sampling point.


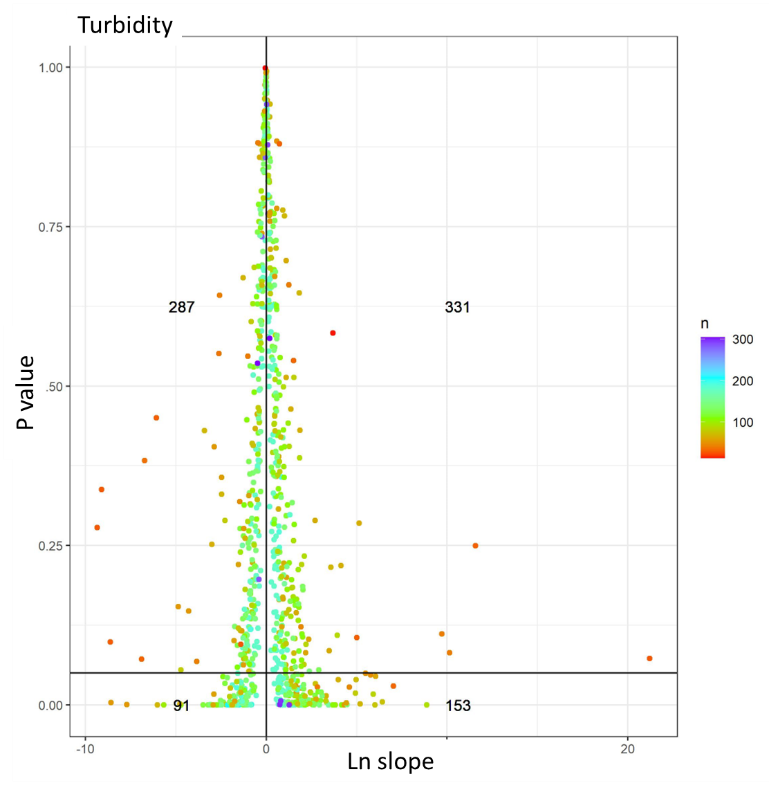


**Fig S8**. Count of sites with either increasing (right of vertical line) or decreasing (left of vertical line) trends in turbidity (log scale) that are significant (below horizontal line) or not (above horizontal line). N refers to the number of samples at each sampling point.


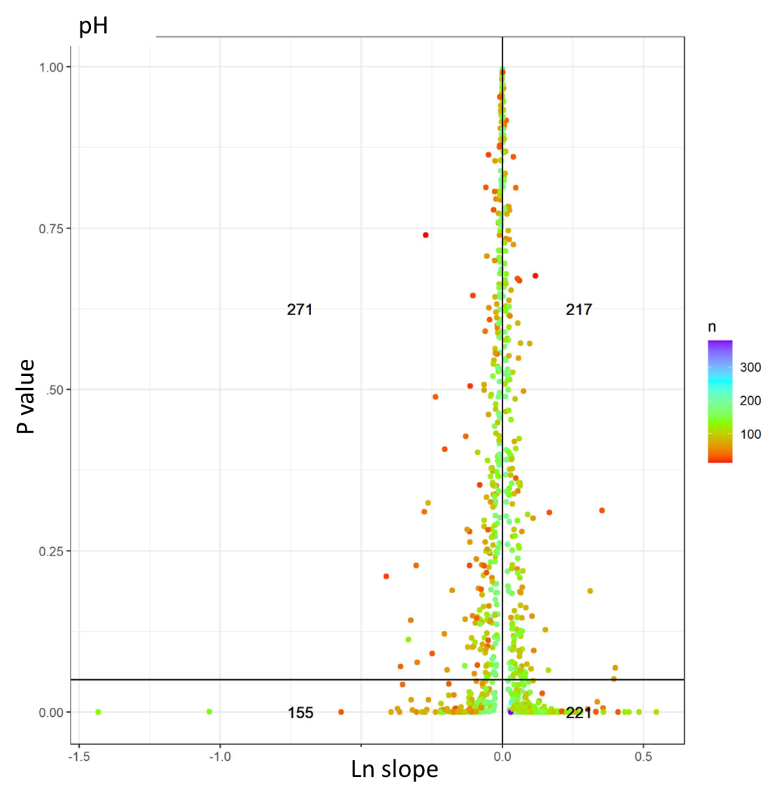


**Fig S9**. Count of sites with either increasing (right of vertical line) or decreasing (left of vertical line) trends in pH (log scale) that are significant (below horizontal line) or not (above horizontal line). Note these data were not used in the analysis but included for reference. N refers to the number of samples at each sampling point.

### Accounting for trends caused by flow.

Many contaminants vary within a year and across years by flow^7^. Hence, flow is often included as a covariate to discern if changes in concentrations are significant^8^. We chose not to adjust trends for flow on the basis that flow is in-turn influenced by climate, and as mentioned above would require prediction of daily climatic variation in the future. However, we checked if accounting for flow would materially affect the standard deviation for all contaminants and hence our calculation of power and sampling frequency. To test this, we developed models with and without a co-variate for flow to predict trends for sites with concentration and flow data available for the same date (n = 360). We applied the same approach to the full dataset containing an additional 410 sites without flow data. We then compared the medians of estimates of standard deviation for both models with and without flow data.

Only small differences were noted between the subset of sites with data and when the analysis was performed on all sites, suggesting that sites with flow data were representative of sites without flow data (Table S5). Although subjective, we considered a ‘material’ difference in standard deviation to be > 10%. Across contaminants, the median percentage difference in median standard deviations were higher (< 10%) for contaminants that were associated with sediment – namely, total nutrients, turbidity, and clarity than not (Table S5). We do recommend that power calculations for these contaminants are made with accompanying flow data. However, there are too few sites (n = 360) with flow data to materially inform the national model. In the absence of additional data, we therefore chose not to include flow-adjusted data in the national models for all contaminants.

**Table S5**. Median standard deviation for the prediction of contaminant concentrations over time (date) with and without flow as a co-variate. Data refers to either a subset of sites (n = 360) where both flow and concentration data exist on the same date or for all sites that include sites without flow data. The percentage difference is the median difference in the standard deviations with and without a co-variate for flow. Percentage differences > 10% are bolded to emphasize the need for flow data to better predict trends in these data.

| Contaminant | Data | Median standard deviation (no flow) | Median standard deviation (with flow) | Median percentage difference in median standard deviation |
| --- | --- | --- | --- | --- |
| NO_3_-N | Subset | 0.868 | 0.797 | 6.5 |
|  | All | 0.874 | 0.797 | 6.8 |
| NH_4_-N | Subset | 0.484 | 0.455 | 2.1 |
|  | All | 0.502 | 0.455 | 3.1 |
| TN | Subset | 0.493 | 0.428 | **12.6** |
|  | All | 0.503 | 0.428 | **12.9** |
| DRP | Subset | 0.474 | 0.446 | 3.4 |
|  | All | 0.474 | 0.446 | 4.0 |
| TP | Subset | 0.630 | 0.510 | **15.5** |
|  | All | 0.630 | 0.510 | **15.9** |
| *E. coli* | Subset | 1.348 | 1.266 | 4.1 |
|  | All | 1.349 | 1.266 | 4.6 |
| Turbidity | Subset | 1.011 | 0.736 | **26.4** |
|  | All | 1.010 | 0.736 | **26.7** |
| pH | Subset | 0.041 | 0.037 | 4.7 |
|  | All | 0.041 | 0.037 | 5.0 |
| Clarity | Subset | 0.806 | 0.572 | **23.5** |
|  | All | 0.817 | 0.572 | **23.6** |

**Fig S10**. Variation in standard deviation for each contaminant with and without flow for sites with data for both concentration and flow in the same date.

## Model performance

Note that the final models (Tables S6 and S7) for each contaminant were produced using data from all 770 sites. We did not separate sites into a training and validation dataset owing to the large number of categorical variables which would have resulted in too few sites in each category to be representative of the diversity of sites in the database. We used the output of the linear model using the standard deviations calculated using a generalised additive model fitted to the variation in contaminant concentrations over time. Note that with GAM models all contaminants except black disk and turbidity exhibited a lower median standard deviation (P<0.05) when using a Mann-Whitney one-way analysis of variance (Fig S11).

**Fig. S11**. Box plots showing the variation in standard deviations using either a linear or generalised additive model (GAM) for the variation in contaminant concentrations over time. Note that all contaminants except black disk and turbidity exhibited a lower median standard deviation (*P*<0.05) when using a Mann-Whitney one-way analysis of variance.

**Table S6**. Terms for significant catchment-specific characteristics and the relative performance as indicated by the coefficient of determination and Akaike Information Criterion included in the linear models produced to predict concentration for each contaminant from sites where concentration over time was predicted using a linear model. Turb = turbidity.

| Parameter | NO_3_-N | NH_4_-N | *E. coli* | DRP | Clarity | Total N | Total P | Turb |
| --- | --- | --- | --- | --- | --- | --- | --- | --- |
| StreamOrde | X | X | X | X | X | X | X | X |
| sinuosity |  | X | X |  | X | X | X | X |
| loc_ind |  | X | X | X | X | X | X | X |
| us_ind | X | X |  | X | X | X | X | X |
| loc_elev | X | X | X | X | X | X | X | X |
| us_elev | X | X | X | X | X | X | X | X |
| loc_slope | X | X | X |  |  | X |  |  |
| us_slope | X | X |  | X | X | X | X | X |
| loc_tmin | X |  |  | X | X | X | X | X |
| us_tmin | X | X | X | X | X | X |  | X |
| local_june | X | X |  | X | X |  | X |  |
| us_june | X | X |  |  |  |  |  |  |
| loc_penpet |  |  |  |  | X | X | X | X |
| us_penpet |  |  |  |  |  | X |  |  |
| loc_rnvar |  | X | X |  | X |  | X | X |
| us_rnvar |  |  |  |  | X | X |  | X |
| loc_rd10 |  | X |  | X |  | X | X | X |
| us_rd10 |  |  |  | X |  |  |  |  |
| loc_rd20 |  | X |  | X | X |  |  | X |
| us_rd20 |  |  |  |  |  |  |  |  |
| loc_rd25 |  |  | X | X |  |  | X |  |
| us_rd25 |  |  |  |  |  |  |  |  |
| loc_rd100 |  |  |  | X |  |  | X | X |
| us_rd100 |  |  |  |  |  |  |  |  |
| loc_phos |  |  |  | X |  |  |  |  |
| us_phos |  |  | X |  |  |  |  |  |
| loc_psize |  |  |  |  | X |  |  |  |
| us_psize | X |  | X |  | X |  |  |  |
| loc_pet |  | X |  | X |  |  |  |  |
| us_pet |  | X |  | X |  |  |  |  |
| loc_mat |  |  |  | X | X |  |  |  |
| us_mat |  |  |  |  | X |  |  |  |
| loc_decs |  |  |  |  | X |  |  |  |
| us_decs |  |  |  |  |  |  |  |  |
| local_twarm |  |  |  |  | X |  |  |  |
| us_twarm |  |  |  |  | X |  |  |  |
| loc_catarea |  |  |  |  |  |  |  |  |
| us_catarea |  |  |  |  |  |  |  |  |
| CLIMATE |  | X | X | X |  | X | X | X |
| SRC_OF_FLW | X |  | X | X | X | X | X | X |
| GEOLOGY |  | X | X | X | X |  | X | X |
| LANDCOVER |  | X | X | X | X | X | X | X |
| NET_POSN |  |  |  |  |  |  |  |  |
| VLY_LNDFRM |  |  |  |  |  |  |  |  |
| SPRING |  |  |  |  |  |  |  |  |
| lcdb_v3_usBare |  | X |  |  | X |  |  | X |
| lcdb_v3_usExoticForest |  |  |  |  |  |  | X |  |
| lcdb_v3_usIntensiveAg | X |  | X | X |  | X | X | x |
| lcdb_v3_usNativeForest |  | X |  |  |  | X |  |  |
| lcdb_v3_usPastoralLight |  |  | X | X |  |  |  |  |
| lcdb_v3_usScrub |  |  |  |  |  |  |  |  |
| lcdb_v3_usUrban |  |  |  | X |  |  |  |  |
| lcdb_v3_usWetland |  |  | X | X |  | X | X |  |
|  |  |  |  |  |  |  |  |  |
| Coefficient of determination | 0.474 | 0.366 | 0.581 | 0.534 | 0.431 | 0.674 | 0.534 | 0.457 |
| Akaike Information Criterion | 2542 | 1816 | 2164 | 1895 | 1299 | 1553 | 1760 | 1873 |

**Table S7**. Terms for significant catchment-specific characteristics and the relative performance as indicated by the coefficient of determination and mean squared error (MSE) included in the linear (L) or random forest (R) models produced to predict standard deviations for each contaminant from unmonitored sites. Turb = turbidity.

| Parameter | NO_3_-N L | NO_3_-N R | NH_4_-N L | NH_4_-N R | *E. coli* L | *E. coli* R | DRP L | DRP R | Clarity L | Clarity R | Total N L | Total N R | Total P L | Total P R | Turb L | Turb R |
| --- | --- | --- | --- | --- | --- | --- | --- | --- | --- | --- | --- | --- | --- | --- | --- | --- |
| StreamOrde | X |  | X |  | X |  |  |  | X |  |  |  | X |  | X |  |
| sinuosity |  |  |  |  |  |  |  |  |  |  |  |  |  |  |  |  |
| us_ind |  | X |  | X | X | X | X |  | X |  | X | X | X | X | X | X |
| us_elev |  | X |  | X |  | X | X | X | X | X |  |  | X |  | X | X |
| us_slope | X | X | X |  | X | X | X | X | X | X | X | X |  | X | X | X |
| us_tmin | X | X | X |  | X | X | X | X | X | X |  | X |  | X |  | X |
| us_june | X | X | X | X |  | X |  |  | X | X | X |  | X | X | X | X |
| us_penpet | X |  | X |  |  |  | X | X | X |  | X | X | X |  | X |  |
| us_rnvar |  | X |  | X |  | X |  | X | X | X |  | X |  | X |  | X |
| us_rd10 |  | X |  | X |  | X |  | X | X | X |  | X |  | X |  | X |
| us_rd20 |  |  |  |  |  |  |  |  |  |  |  |  |  |  |  |  |
| us_rd25 |  | X |  | X |  | X |  | X |  | X |  | X |  | X |  | X |
| us_rd100 |  |  |  | X |  |  |  |  |  | X | X |  |  |  | X |  |
| us_phos | X | X | X | X |  | X |  | X | X | X | X | X |  | X |  | X |
| us_psize | X | X | X | X | X | X | X | X | X | X | X | X | X | X | X | X |
| us_pet | X |  | X | X |  | X | X | X | X | X | X |  | X | X | X | X |
| us_mat |  | X |  | X |  | X |  |  | X | X |  | X | X |  | X | X |
| us_decs | X | X | X | X |  | X |  | X | X | X |  | X |  | X |  | X |
| us_twarm |  | X |  |  |  | X |  | X | X | X |  | X |  |  |  | X |
| us_catarea |  | X |  | X |  | X |  | X | X | X |  | X |  | X |  | X |
| CLIMATE | X | X | X |  |  |  | X |  | X |  | X |  |  |  |  |  |
| SRC_OF_FLW |  |  |  |  | X |  | X |  | X |  |  |  | X |  | X |  |
| GEOLOGY | X | X | X | X |  | X | X | X | X | X | X | X | X | X | X | X |
| LANDCOVER | X |  | X | X | X |  | X | X | X |  | X |  | X |  | X |  |
| NET_POSN | X |  | X |  |  |  |  |  |  |  |  |  |  |  |  |  |
| VLY_LNDFRM |  |  |  |  |  |  |  |  |  |  |  |  |  |  |  |  |
| SPRING |  |  |  |  |  |  |  |  |  |  |  |  |  |  |  |  |
| lcdb_v3_usBare |  | X |  | X |  | X |  | X |  | X |  | X | X | X | X | X |
| lcdb_v3_usExoticForest | X | X | X | X |  | X | X | X | X | X | X | X | X | X | X | X |
| lcdb_v3_usIntensiveAg | X | X | X | X | X | X | X | X | X |  |  | X |  | X | X | X |
| lcdb_v3_usNativeForest |  | X |  | X |  | X |  | X | X |  |  |  |  |  |  | X |
| lcdb_v3_usPastoralLight |  |  |  |  |  |  | X | X | X | X | X |  |  |  |  |  |
| lcdb_v3_usScrub |  | X |  | X |  | X |  | X | X | X |  |  |  |  |  |  |
| lcdb_v3_usUrban |  | X |  | X |  | X |  | X |  |  |  |  |  |  |  |  |
| lcdb_v3_usWetland |  | X |  | X |  | X |  | X |  |  |  |  |  |  | X | X |
|  |  |  |  |  |  |  |  |  |  |  |  |  |  |  |  |  |
| Coefficient of determination | 0.30 | 0.46 | 0.44 | 0.51 | 0.23 | 0.33 | 0.31 | 0.44 | 0.60 | 0.66 | 0.28 | 0.46 | 0.45 | 0.54 | 0.53 | 0.67 |
| MSE | 0.096 | 0.070 | 0.029 | 0.028 | 0.054 | 0.044 | 0.024 | 0.017 | 0.030 | 0.023 | 0.019 | 0.014 | 0.030 | 0.024 | 0.063 | 0.042 |

## Comparing error at different sampling frequencies

Since our power calculations were completed using the standard deviations from monthly observations it is plausible to suggest that they may not apply when predicting power at greater sampling frequencies. While some work has shown that mean concentrations derived from annual or monthly data are just as variable as those collected days or hours apart^25^, most studies show the opposite ^26-28^. To test the validity of monthly data for predicting high frequency sampling we collected data from sites from three Regional Councils that had NO_3_-N and turbidity observations measured via *in-situ* probes at a 30-minute interval (Table S8). Nitrate-N and turbidity likely represent the spectrum of mobility in our contaminants (i.e., readily leached with water (NO_3_-N) or attached and eroded to soil particles – all other contaminants). The standard deviations for both analytes derived from the fit of a GAM were generally smaller for samples taken on a 30-minute, daily or weekly interval compared to monthly sub-sampling. A high standard deviation avoids the risk of underestimating the number of samples required to detect change.

**Table S8**. Name, location, and length of data record for each high-resolution data site.

| Contaminant / Site name | Long | Lat | Years of record | 30-min | Daily | Weekly | Monthly |
| --- | --- | --- | --- | --- | --- | --- | --- |
| Nitrate-N |  |  |  |  |  |  |  |
| Aparima River | -45.1766 | 170.8810 | 6.8 | 0.276 | 0.268 | 0.301 | 0.391 |
| Hurunui River | -45.1180 | 170.8274 | 1.1 | 0.130 | 0.127 | 0.118 | 0.137 |
| Kaiapoi River | -44.9674 | 168.8130 | 3.0 | 0.648 | 0.662 | 0.680 | 0.797 |
| Kakanui River at Gemmels | -45.4252 | 170.6636 | 5.9 | 0.233 | 0.223 | 0.071 | 0.071 |
| Kakanui River at McCones | -45.4758 | 170.7556 | 2.9 | 0.270 | 0.722 | 0.240 | 0.262 |
| Mill Creek | -43.4245 | 172.5653 | 3.7 | 0.035 | 0.035 | 0.043 | 0.067 |
| Shag River at Craig Road | -42.8986 | 173.0967 | 4.2 | 0.211 | 0.205 | 0.203 | 0.231 |
| Shag River at Shakey Bridge | -44.0683 | 171.6518 | 4.2 | 0.148 | 0.123 | 0.139 | 0.164 |
| Windermere drain | -46.2856 | 168.0842 | 7.3 | 2.941 | 2.885 | 0.070 | 3.570 |
| Turbidity |  |  |  |  |  |  |  |
| Mangahao | -39.3884 | 174.4666 | 1.2 | 0.025 | 0.051 | 0.026 | 0.030 |
| Mangati River | -39.0349 | 174.1503 | 4.1 | 0.027 | 0.027 | 0.022 | 0.025 |
| Tangahoe River | -39.6297 | 174.3488 | 0.4 | 0.067 | NA | NA | NA^1^ |
| Waingongoro River | -39.5765 | 174.2097 | 1.5 | 0.072 | 0.160 | 0.104 | 0.109 |
| Wakapuaka River | -41.2156 | 173.3955 | 4.9 | 0.070 | 0.071 | 0.071 | 0.076 |
| Horokiri Stream | -41.0793 | 174.9262 | 7.5 | 0.066 | 0.065 | 0.077 | 0.081 |
| Pauatahanui Stream | -41.1169 | 174.9234 | 7.5 | 0.042 | 0.041 | 0.050 | 0.051 |
| Porirua Stream | -41.1409 | 174.8430 | 7.1 | 0.041 | 0.039 | 0.042 | 0.043 |
| Manawatu River | -40.2395 | 176.1164 | 7.9 | 0.083 | 0.079 | 0.069 | 0.078 |
| Mangaehu River | -40.4361 | 175.7917 | 3.1 | 0.019 | 0.018 | 0.025 | 0.027 |
| Mangatainoka River | -40.4486 | 175.8320 | 8.4 | 0.029 | 0.027 | 0.029 | 0.029 |
| . Ohura River | -38.9175 | 175.0324 | 11.4 | 0.038 | 0.037 | 0.038 | 0.040 |
| Pohangina River | -40.2246 | 175.7830 | 8.7 | 0.066 | 0.064 | 0.058 | 0.060 |
| Rangitikei River | -39.8094 | 175.8081 | 8.5 | 0.041 | 0.040 | 0.042 | 0.045 |
| Tiraumea River | -40.4671 | 175.9228 | 7.9 | 0.061 | 0.058 | 0.058 | 0.062 |

^1^ A meaningful GAM could not be produced owing to the short period of record

## Discrete sampling monitoring costs

Annual cost estimates (in New Zealand dollars: 1NZD = 0.60 USD as of September 2022) included capital expenditure and operational expenditure.

For capital expenditure (Capex) we included the purchase of a vehicle ($60,000) and a hand-held multimeter ($2,500) for field measurement of pH, temperature, and dissolved oxygen. Equipment for estimating clarity via the black disc method was also included in capital expenditure ($340 NZD). All capital cost items were renewed every 5-years. Depreciation was not accounted for.

For operational expenditure (Opex), laboratory analytical costs were calculated as the average of the cost provided by the three commercial laboratories for each contaminant. These were $19.35, 9.34, 11.79, 15.51, 24.85, 19.55, 24.69, and 35.32 NZD for total suspended sediments, turbidity, nitrate-N, ammoniacal-N, total P, dissolved reactive P, total P and *E. coli*, respectively. Other operational costs included mileage (at $0.71 NZD per kilometre), personal protective equipment (PPE) and staff time.

Costs for Capex (assuming each authority purchased separate capital equipment), staff time, personnel protection equipment (PPE) and mileage was summed across all each authority and divided by the number sites in the monitoring programme (varying from 50-150 sites per Regional Authority) and by the number of monitoring events per annum (12) to obtain an average cost per site and per monitoring event (‘site visit’) (Table S6). Contaminant-specific costs were then calculated by adding the contaminant cost to the average cost per site visit. This provides costs associated with obtaining one data point for each individual water quality contaminant or field measurement. These individual costs should not be added to calculate the total costs associated with a monitoring regime involving several contaminants or field measurements.

To facilitate a comparative analysis of costs at varying frequency for each water quality contaminant, the cost of collecting one data point for a given variable was calculated assuming only that variable was collected at each monitoring event. For example, the cost of collecting one Total Nitrogen datapoint was calculated as the sum of vehicle, Sampling Equipment, Mileage, PPE, staff time and analytical cost for one TN analysis.

**Table S9**. Breakdown of costs associated with the analysis of water quality contaminants.

| Cost ($NZD) for water quality contaminant (per measurement and visit) | Capex | Opex | Labour | Laboratory | Total |
| --- | --- | --- | --- | --- | --- |
| Suspended sediment | 8.65 | 18.91 | 140.32 | 19.35 | 187.23 |
| Turbidity | 8.65 | 18.91 | 140.32 | 9.34 | 177.22 |
| Nitrate-N | 8.65 | 18.91 | 140.32 | 11.79 | 179.67 |
| Ammoniacal-N | 8.65 | 18.91 | 140.32 | 15.51 | 183.39 |
| Total Nitrogen | 8.65 | 18.91 | 140.32 | 24.85 | 192.72 |
| Dissolved Reactive Phosphorus | 8.65 | 18.91 | 140.32 | 19.55 | 187.42 |
| Total Phosphorus | 8.65 | 18.91 | 140.32 | 24.69 | 192.57 |
| *E. coli* | 8.65 | 18.91 | 140.32 | 35.32 | 203.20 |
| Dissolved Oxygen | 1.30 | 20.76 | 140.32 | - | 133.96 |
| Temperature | 1.30 | 20.76 | 140.32 | - | 133.96 |
| pH | 1.30 | 20.76 | 140.32 | - | 133.96 |
| Clarity | 0.41 | 18.91 | 140.32 | - | 132.76 |

**References**

1 LAWA. Land, Air, Water, Aotearoa. (2015). < <http://www.lawa.org.nz>>.

2 Ministry for the Environment. *Freshwater classification system: River environment classification* <<https://www.mfe.govt.nz/environmental-reporting/about-environmental-reporting/classification-systems/fresh-water.html>> (2013).

3 Snelder, T. H., Biggs, B. J. F. & Woods, R. A. Improved eco-hydrological classification of rivers. *River Res. Appl.* **21**, 609-628 (2005).

4 Whitehead, A. Spatial modelling of river water-quality state. Incorporating monitoring data from 2013 to 2017. 41 (NIWA, Christchurch, New Zealand, 2018).

5 Manaaki Whenua Landcare Research. *LCDB v5.0 - Land Coiver Database version 5.0*, <<https://lris.scinfo.org.nz/layer/104400-lcdb-v50-land-cover-database-version-50-mainland-new-zealand/>> (2020).

6 Snelder, T. H., Larned, S. T., Fraser, C. & De Malmanche, S. Effect of climate variability on water quality trends in New Zealand rivers. *Mar. Freshwat. Res.* **73**, 20-34 (2022).

7 Cartwright, I., Morgenstern, U. & Hofmann, H. Concentration versus streamflow trends of major ions and tritium in headwater streams as indicators of changing water stores. *Hydrol. Process.* **34**, 485-505 (2020).

8 Time Trends Software v. 3.0 (Natonal Institute of Water and Atmospheric Research, Christchurch, New Zealand, 2009).
